# Supplementary material for: Habitat selection in natural and human-modified landscapes by capybaras (Hydrochoerus hydrochaeris), an important host for Amblyomma sculptum ticks
Source: PLoS One. 2020 Aug 20;15(8):e0229277. doi: 10.1371/journal.pone.0229277 (PMC7444575; doi:10.1371/journal.pone.0229277)
Supplement: S1 Appendix — Methods on how habitats of studied capybaras were classified using high-resolution satellite imagery and random forest algorithm. (DOCX) [file pone.0229277.s001.docx]

# S1 Appendix

We used high-resolution imagery from 8-band WorldView-2 satellite (DigitalGlobe, Inc.) to perform land cover classification using Random Forest algorithm (see [1] for further information about the use of Random Forest on land cover classification).

We generated two tasseled-cap transformed layers (brightness and wetness/shadows) using Kauth-Thomas transformation coefficients [2]. Following the equations from a previous work [3], we also created two texture metrics layers (dissimilarity and second-moment) through NDVI scenes. The eight bands of WorldView-2 scenes, NDVI, tasseled cap transformed layers, and texture metrics were used as input to the land cover classification (Table A).

Table A. Input data for land cover classification (8-bands WorldView-2 scene, NDVI, tasseled cap layers and texture metrics). Land cover was performed using Random Forest classification of high-resolution imagery (WorldView-2; 2-m resolution).

| *Input data* | | | | *Layer description* |
| --- | --- | --- | --- | --- |
| *Worldview-2 satellite band* | Band 1 | Coastal Blue | Band 1 (0.400-0.450 *µm*) | |
|  | Band 2 | Blue | Band 2 (0.450-0.510 *µm*) | |
|  | Band 3 | Green | Band 3 (0.510-0.580 *µm*) | |
|  | Band 4 | Yellow | Band 4 (0.585-0.625 *µm*) | |
|  | Band 5 | Red | Band 5 (0.630-0.690 *µm*) | |
|  | Band 6 | Red Edge | Band 6 (0.705-0.745 *µm*) | |
|  | Band 7 | NIR* 1 | Band 7 (0.770-0.895 *µm*) | |
|  | Band 8 | NIR* 2 | Band 8 (0.860-1.040 *µm*) | |
| *Vegetation Index* | NDVI | | Layer generated following the equation in [4]: $\frac{{band 7}_{NIR1}- {band 5}_{RED}}{{band 7}_{NIR1}+ {band 5}_{RED}}$ | |
| *Tasseled cap transformation* | Brightness | | Layer of terrestrial soil reflectance. | |
|  | Wetness | | Layers related to terrestrial surface wetness. | |
| *Texture metrics* | Dissimilarity | | $\sum_{n=0}^{N-1} n\left\{ \sum_{i=1}^{N} \sum_{j=1}^{N} p(i,j) \right\}$ | |
|  | Angular-second moment | | $\sum_{i} \sum_{j} \left\{ p(i,j) \right\}^{2}$ | |

*Near Infrared

We classified nine WorldView-2 scenes containing all study areas across natural and human-modified landscapes (Table B). A total of 1531 testing polygons (ranging from 102 to 252 by scene) were digitized based on visual interpretation of satellite imagery using QGIS 2.18.9 [5], a relatively common technique [6, 7, 8] mainly used for high-resolution satellite imagery. We divided polygons in calibration (70%), used as input for the land cover classification, and validation (30%), used to evaluate classification accuracy. We used four land cover classes (forest, water, grasses/shrubs and bare soil) to perform classification across natural landscapes, and five classes in human-modified landscapes (those previously mentioned and a class containing roads/settlements).

We accessed prediction map’s accuracy was accessed using Kappa’s Coefficient [9] and overall accuracy. Overall accuracy results ranged from 0.95 to 1 across natural landscapes ($\bar{x}=0.97;n=3$) and from 0.84 to 0.99 in human-modified landscapes ($\bar{x}=0.94;n=6$). Kappa coefficient ranged from 0.93 to 1 across natural landscapes ($\bar{x}=0.96;n=3$) and from 0.78 to 0.98 across human-modified landscapes ($\bar{x}=0.92;n=6$). We performed analyzes using ‘*RStoolbox’* package [10] in R statistical environment [11]. We also applied a post- classification filter (3x3 window size) using ‘*focal’* function in ‘*raster’* package [12] to reduce ‘salt-and-pepper’ noise generate by per-pixel classifiers such as Random Forest [13]. The land cover classifications were then visually inspected for misclassifications and corrected when necessary.

Table B. Land cover classification accuracy assessment for WorldView-2 scenes across natural and human-modified landscapes. Overall accuracy, Kappa coefficient and the number of testing polygons are displaced by study area.

| *Study Area** | *Worldview-2 Scene Date* | *Overall Accuracy* | *Kappa Coefficient* | *Testing Polygons* |
| --- | --- | --- | --- | --- |
| Ingá/São Jose (NL) | March 12, 2017 | 0.9751 | 0.9660 | 115 |
| Ipanema (NL) | November 24, 2016 | 0.9980 | 0.9973 | 102 |
| Poconé (NL) | September 01, 2016 | 0.9508 | 0.9310 | 138 |
| Americana (HML) | August 28, 2017 | 0.9504 | 0.9359 | 187 |
| Araras (HML) | August 14, 2016 | 0.9147 | 0.8915 | 252 |
| Piracicaba (HML) | August 28, 2017 | 0.9759 | 0.9695 | 246 |
| Pirassununga (HML) | August 16, 2017 | 0.9874 | 0.9839 | 210 |
| Ribeirão Preto (HML) | August 09, 2017 | 0.9575 | 0.9451 | 158 |
| São Paulo (HML) | August 06, 2016 | 0.8432 | 0.7773 | 123 |

*NL = natural landscape; HML = human-modified landscape

**References**

1. Gislason PO, Benediktsson JA, Sveinsson JR. Random forests for land cover classification. Pattern Recogn Lett. 2006; 27: 294-300.
2. Yarbrough LD, Navulur K, Ravi R. Presentation of the Kauth–Thomas transform for WorldView-2 reflectance data. Remote Sens Lett. 2014; 5: 131- 138.
3. Haralick RM, Shanmugam K. Textural features for image classification. IEEE T Syst Man Cyb. 1973; 6: 610-621.
4. Tucker CJ, Sellers PJ. Satellite remote sensing of primary production. Int J Remote Sens. 1986; 7: 1395-1416.
5. QGIS. An Open Source Geographic Information System. QGIS. 2017.
6. Huang C, Kim S, Song K, Townshend JR, Davis P, Altstatt A, et al. Assessment of Paraguay's forest cover change using Landsat observations. Global Planet Change. 2009; 67: 1-12.
7. Stow D, Lopez A, Lippitt C, Hinton S, Weeks J. Object‐based classification of residential land use within Accra, Ghana based on QuickBird satellite data. Int J Remote Sens. 2007; 28: 5167-5173.
8. Vanonckelen S, Lhermitte S, van Rompaey A. The effect of atmospheric and topographic correction methods on land cover classification accuracy. Int J Appl Earth Obs. 2013; 24: 9-21.
9. Cohen JA. Coefficient of agreement for nominal scales. Educ Psychol Meas. 1960; 20: 37-46.
10. Leutner B, Horning N. RStoolbox: Tools for Remote Sensing Data Analysis. CRAN–Package version 01; 2016.
11. R Core Team. R: A language and environment for statistical computing. R Foundation for Statistical Computing; 2013.
12. Hijmans RJ. Package ‘raster’. R package version 02; 2014.
13. Lu D, Weng Q. A survey of image classification methods and techniques for improving classification performance. Int J Remote Sens. 2007; 28: 823-870.
