# Supplementary material for: Habitat selection in natural and human-modified landscapes by capybaras (Hydrochoerus hydrochaeris), an important host for Amblyomma sculptum ticks
Source: PLoS One. 2020 Aug 20;15(8):e0229277. doi: 10.1371/journal.pone.0229277 (PMC7444575; doi:10.1371/journal.pone.0229277)
Supplement: S2 Appendix — We performed sensitivity analysis to set the number of random points per ‘use’ point to our habitat selection models. (DOCX) [file pone.0229277.s002.docx]

# S2 Appendix

Figure A. Sensitivity Analysis performed for study areas across natural and human-modified landscapes following [1]. São José, Ingá, Ipanema and Poconé were located in natural landscapes, all other study areas were located across human-modified landscapes. The *x* axis represents the number of availability points by GPS-location (1, 2, 3, 5, 10, 20, 30 and 50). The *y* axis provides the generalized linear model coefficients. In this study we decided to use 30 availability-points by GPS-location.


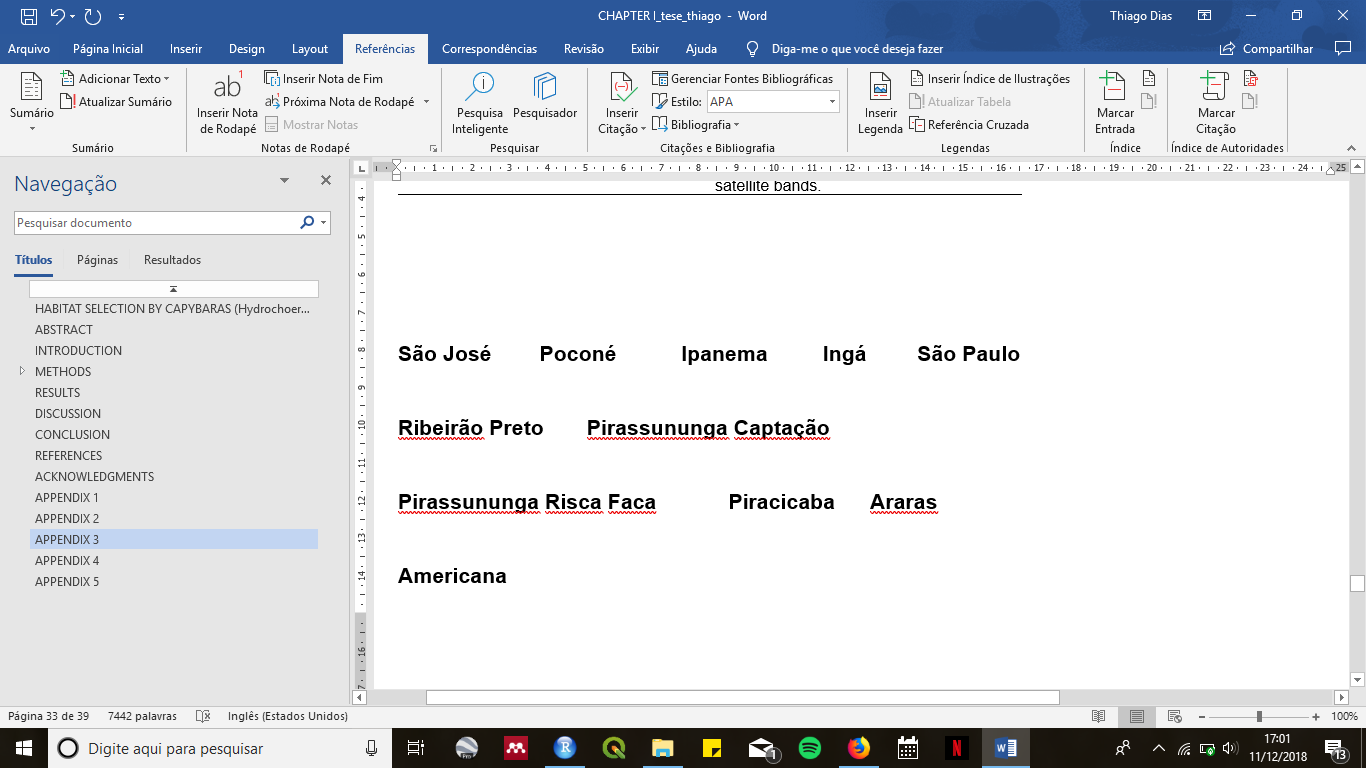

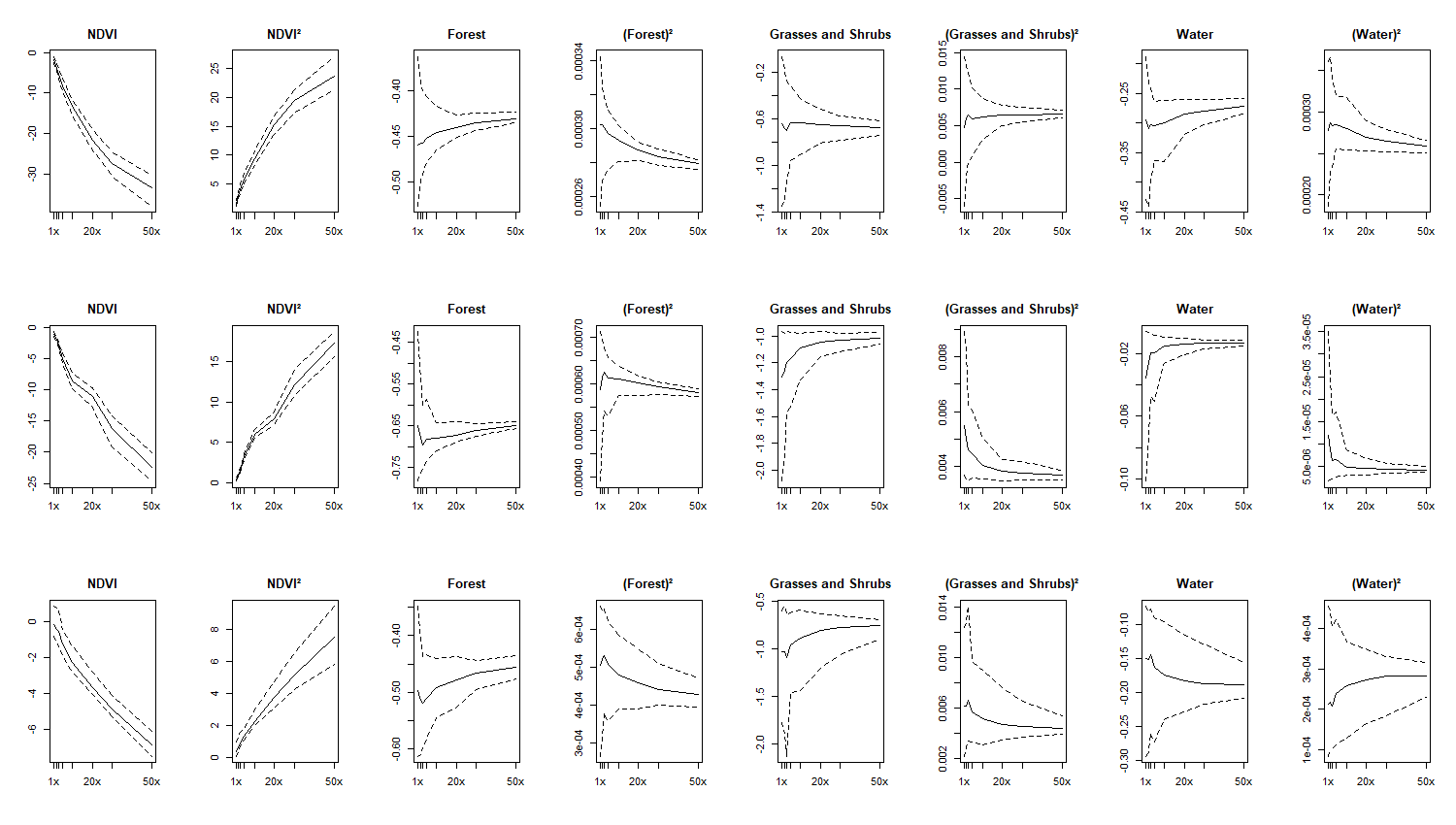


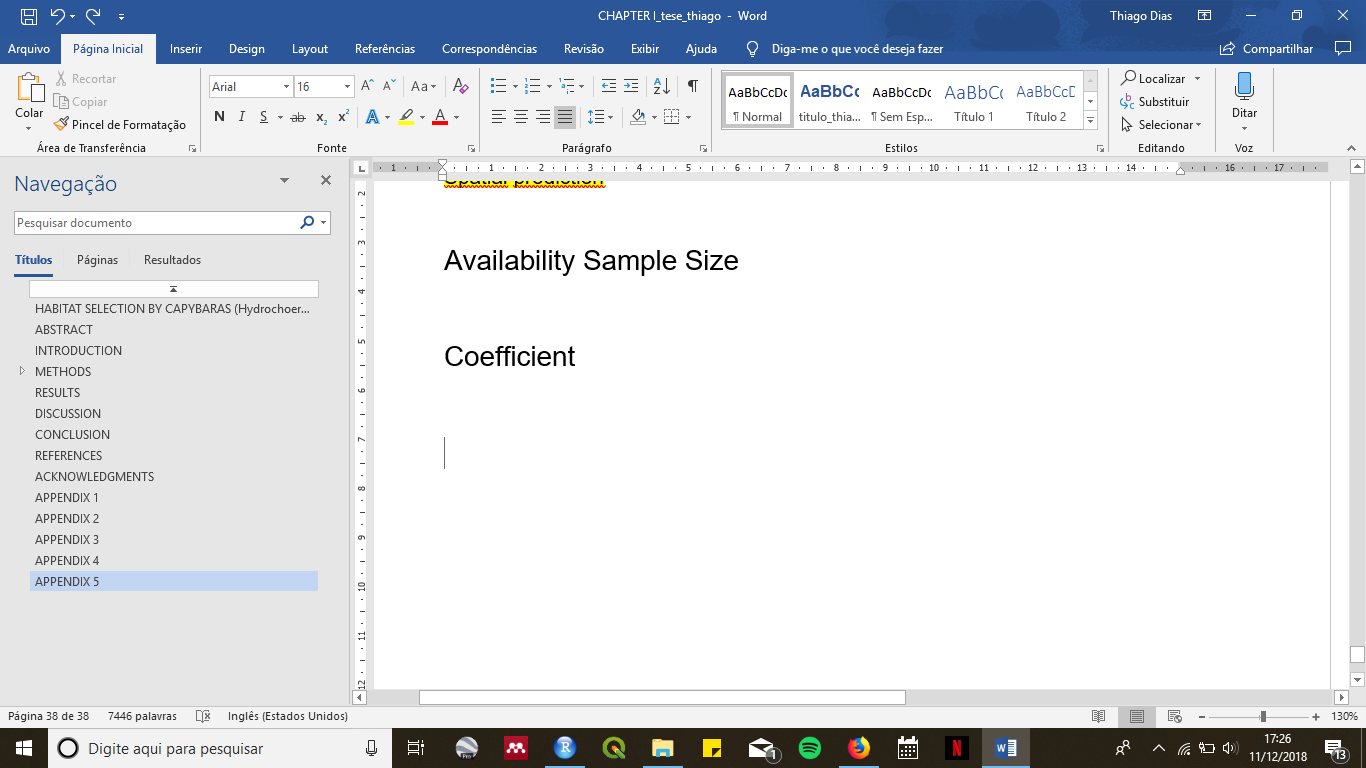

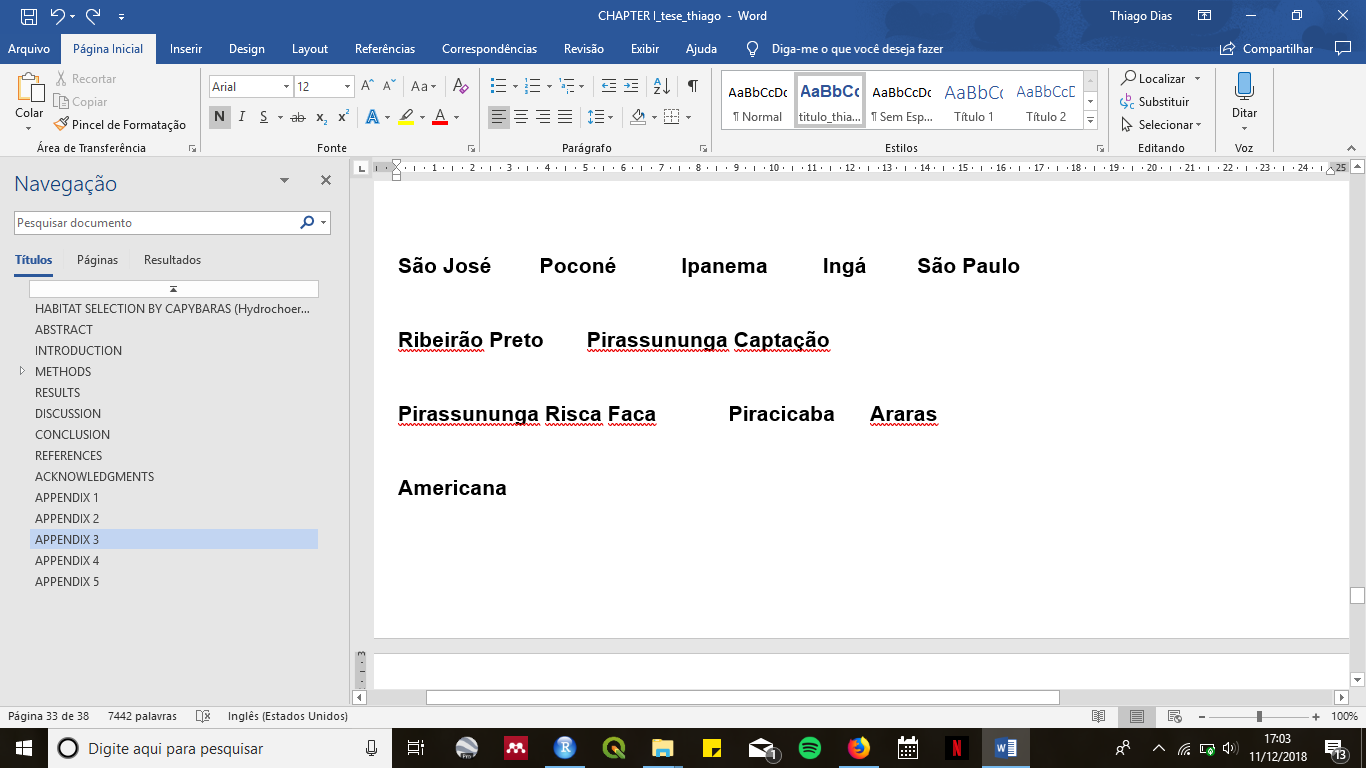

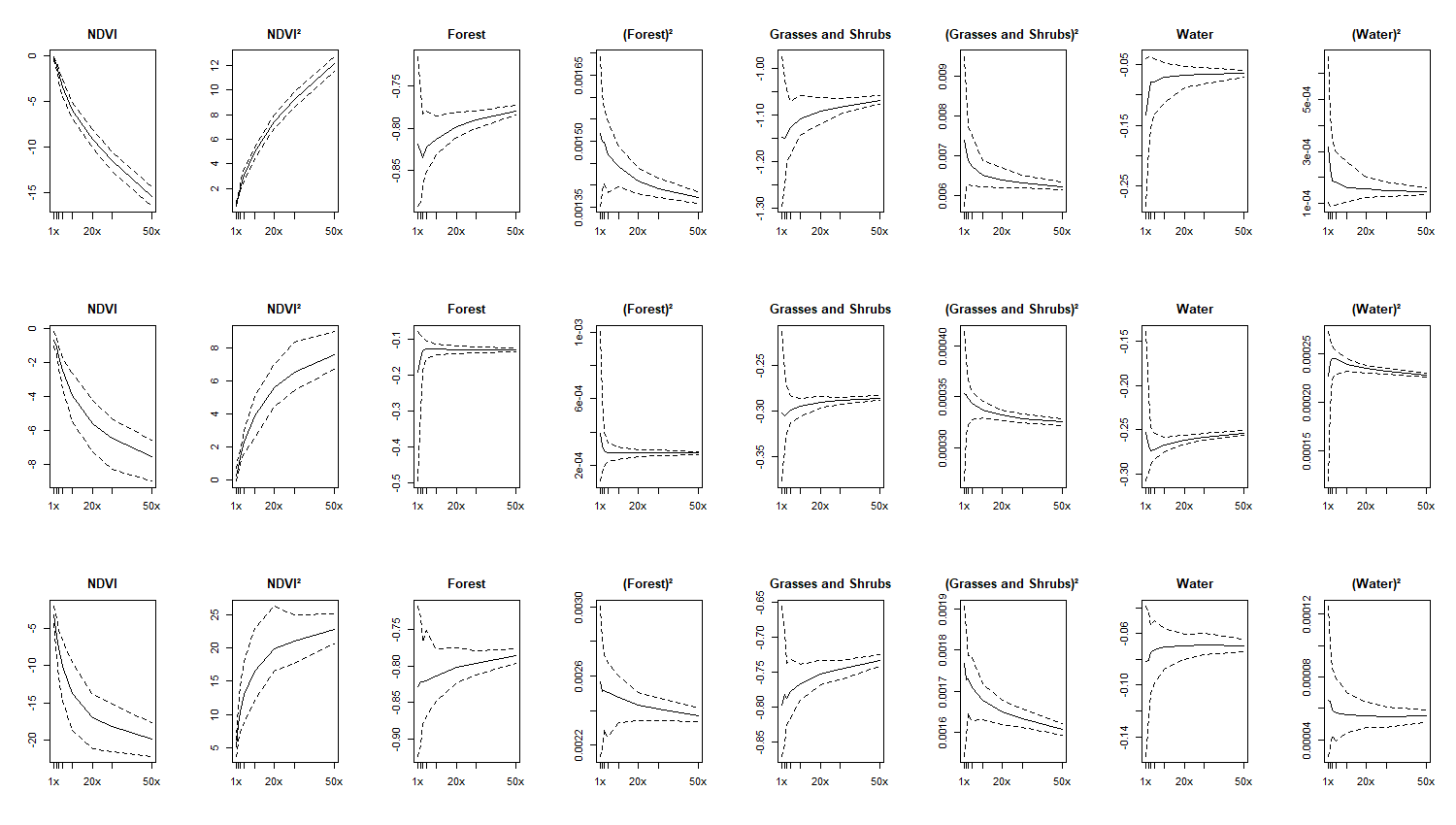


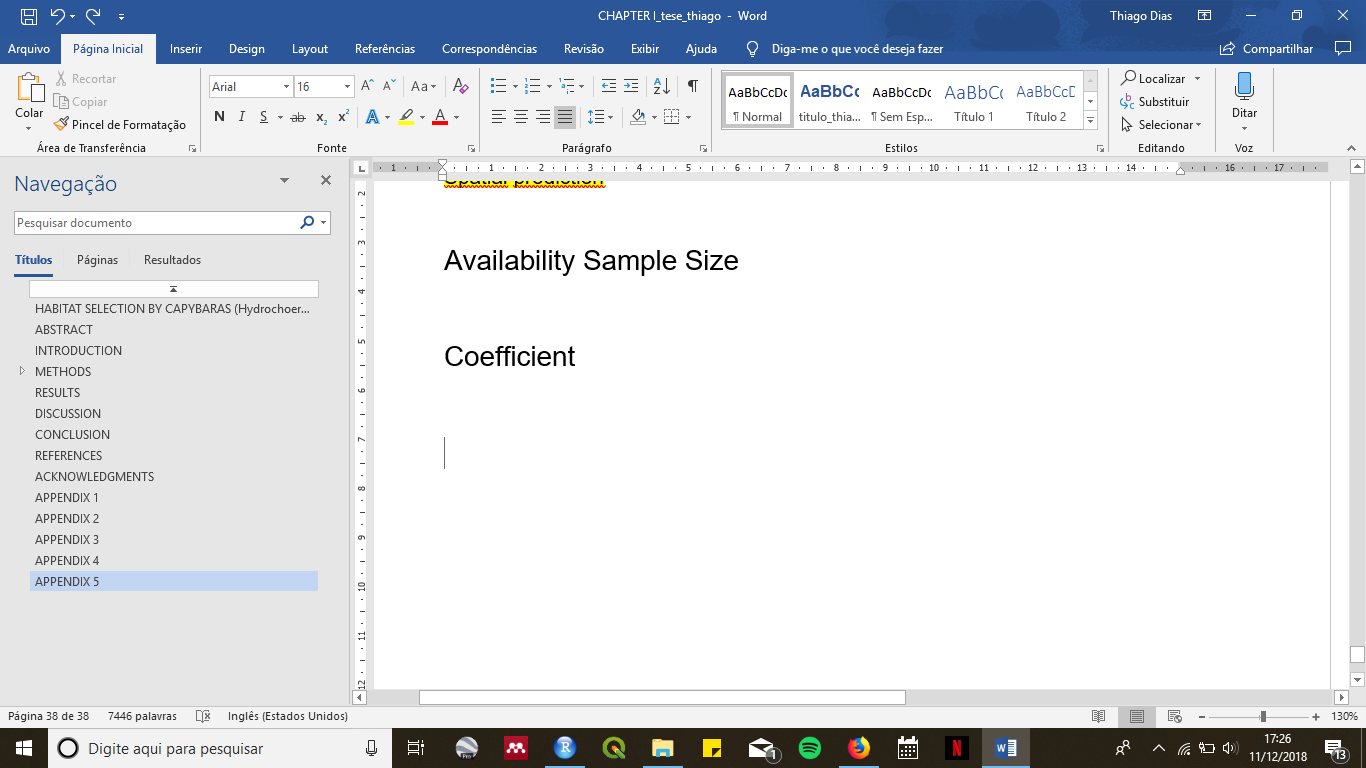

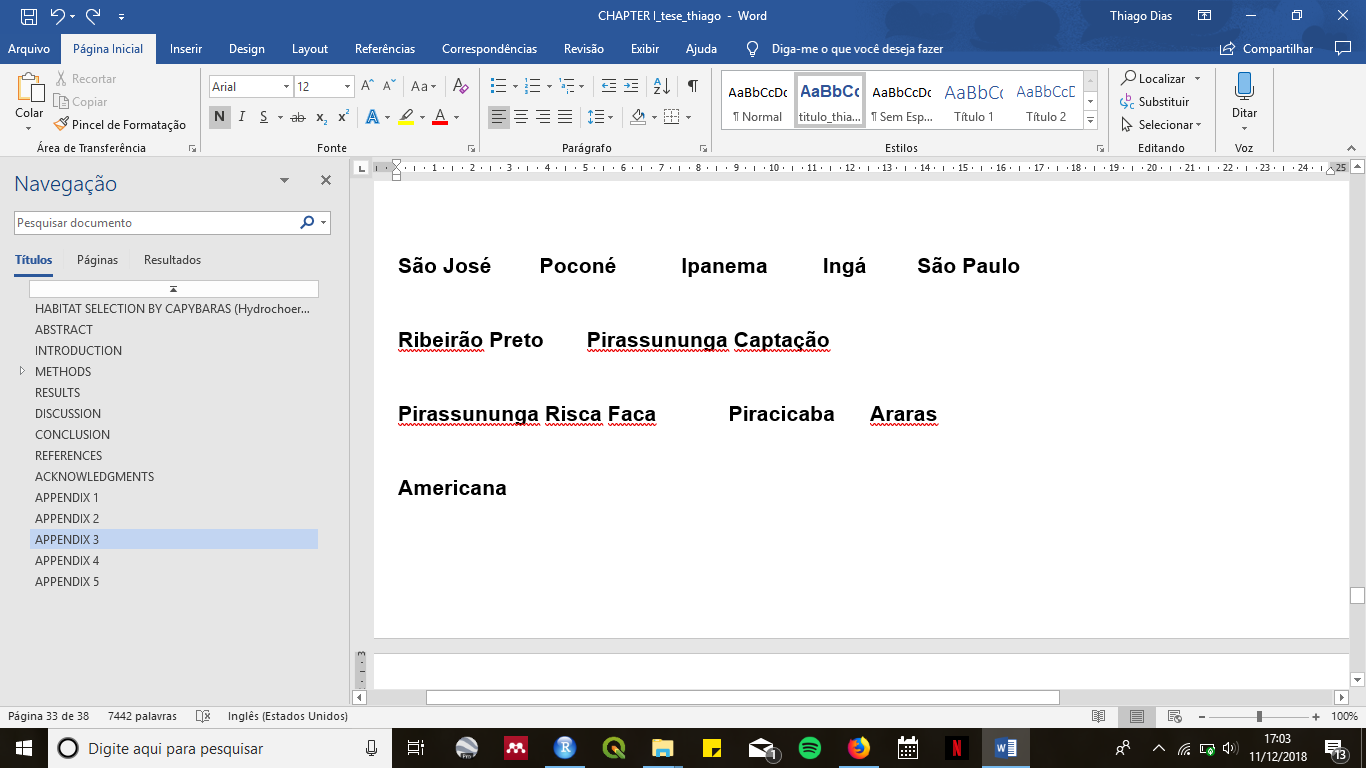

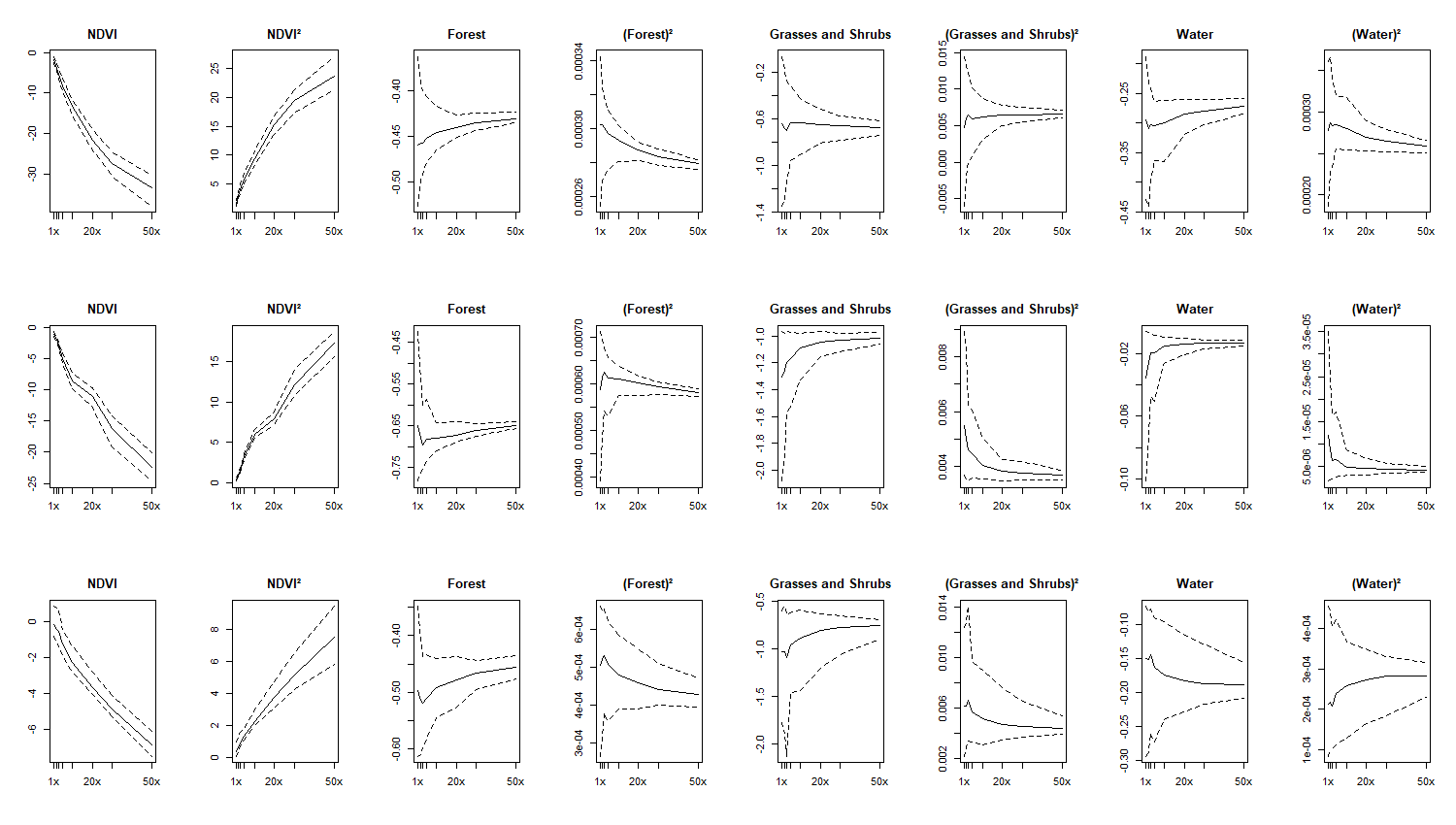


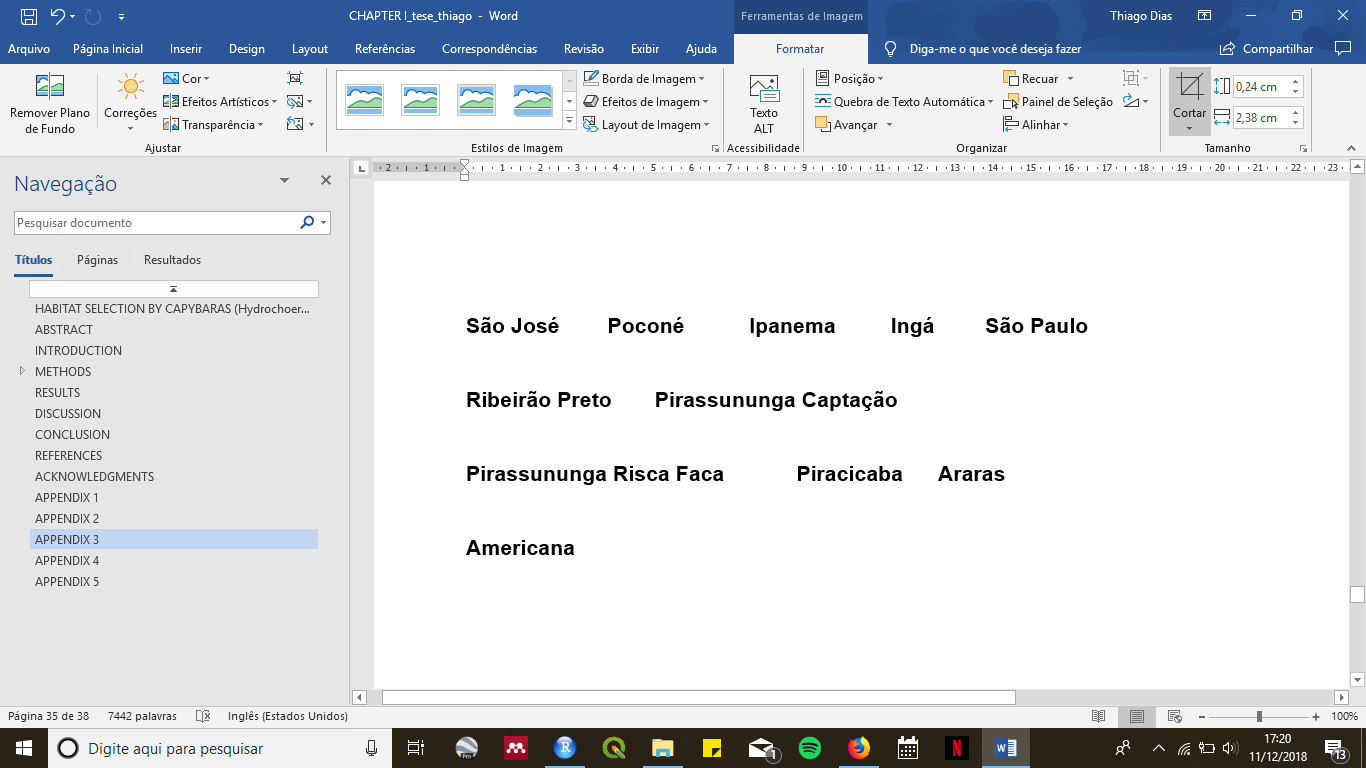

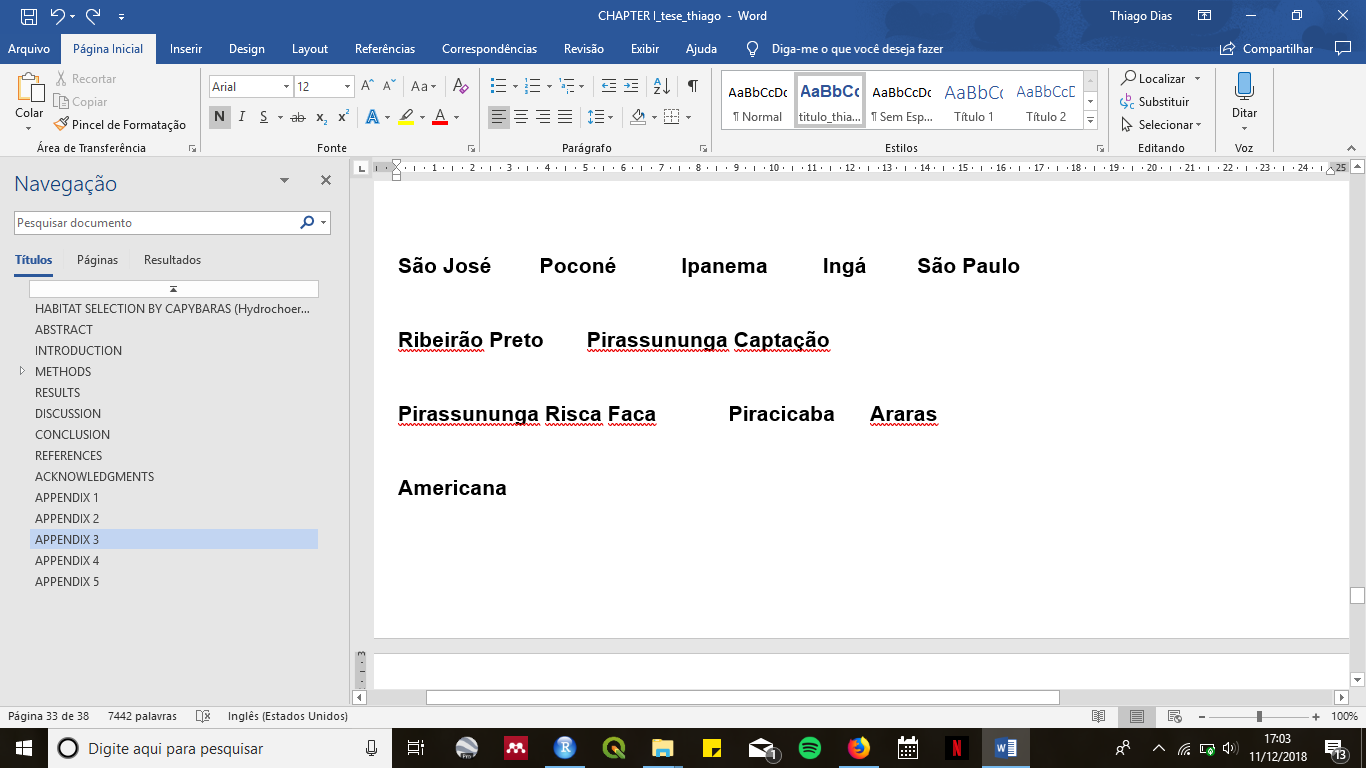

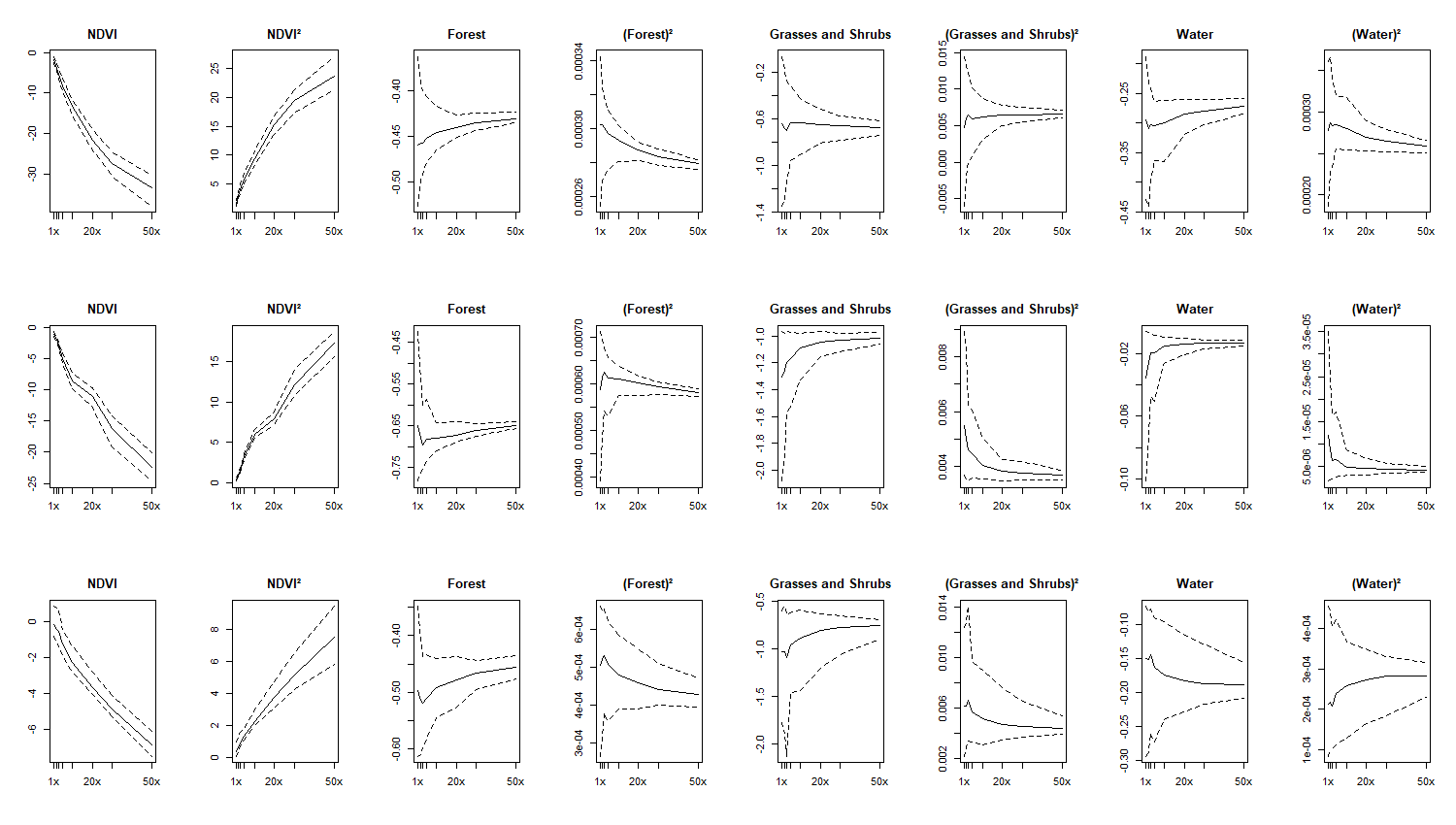

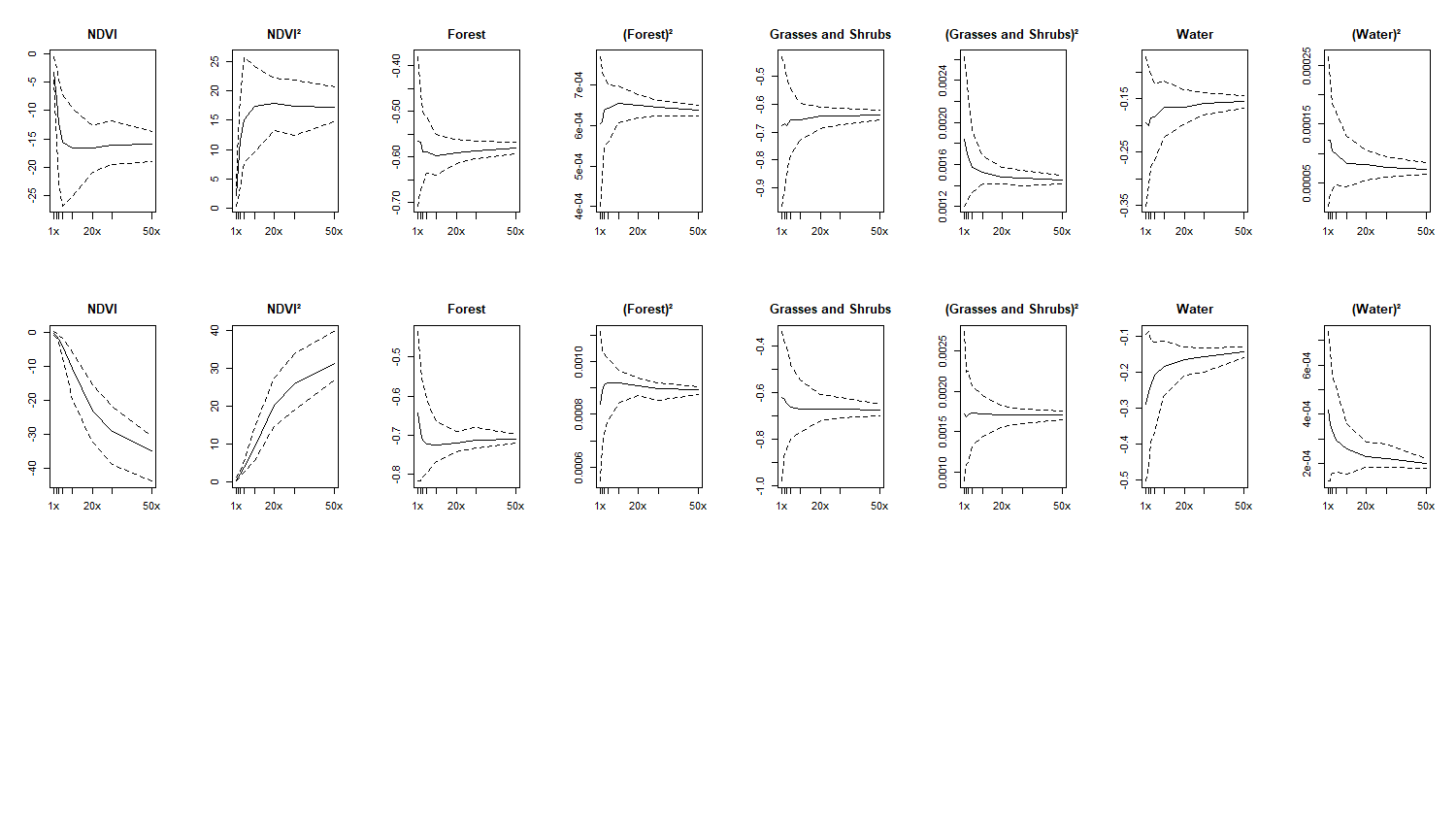


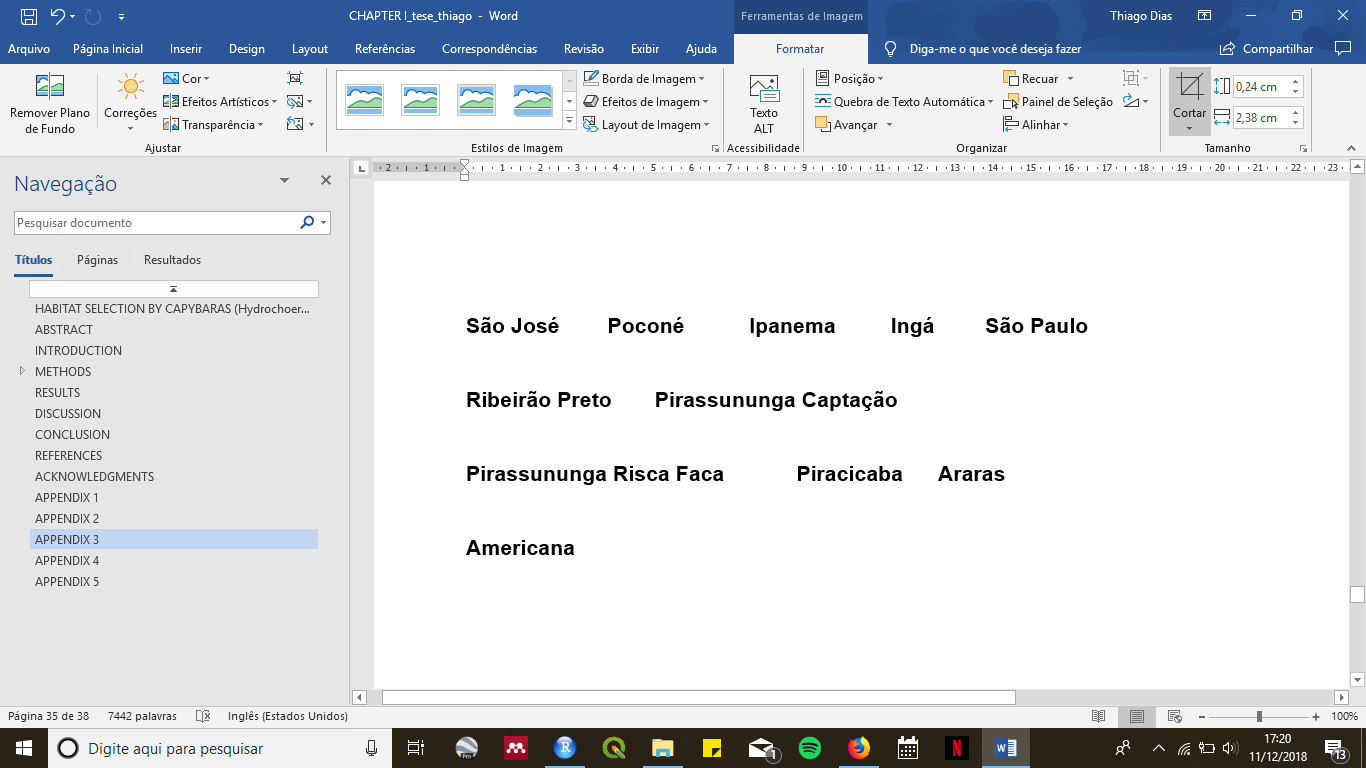

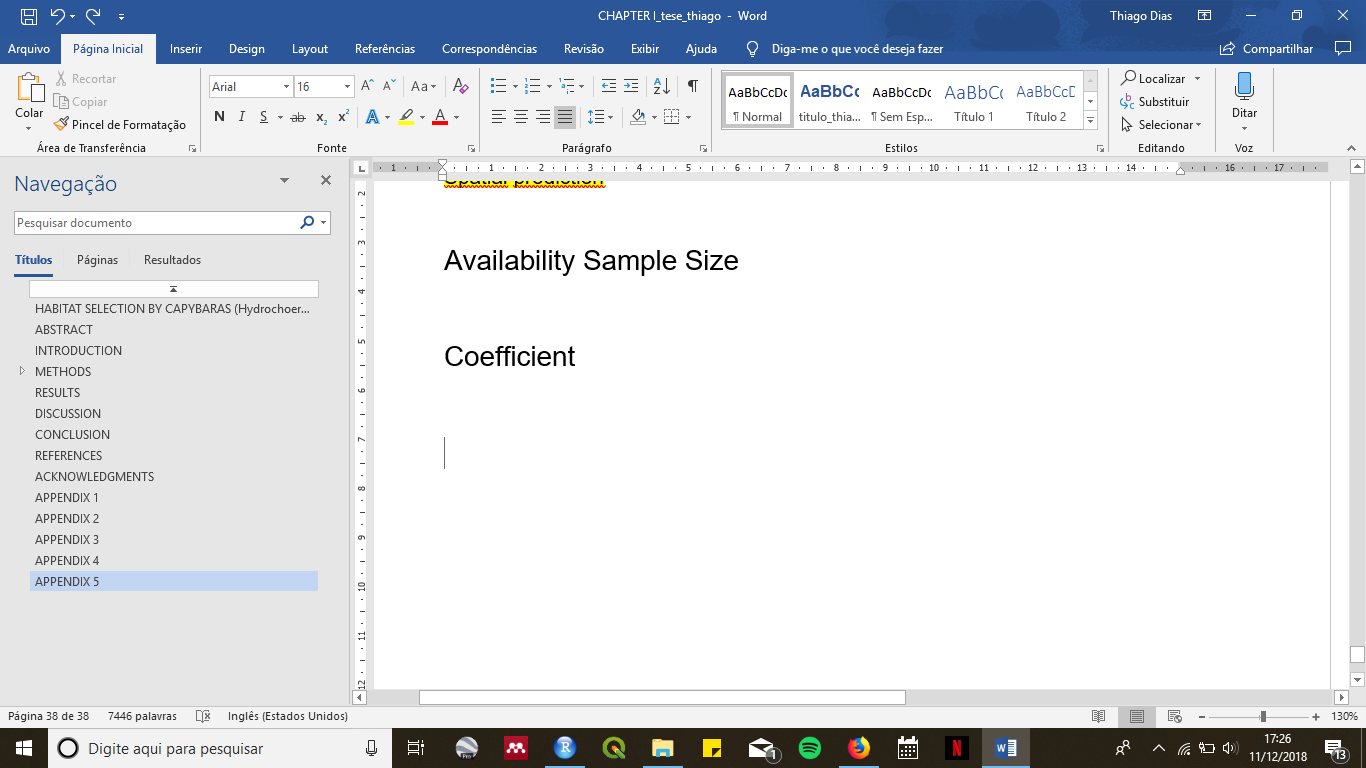

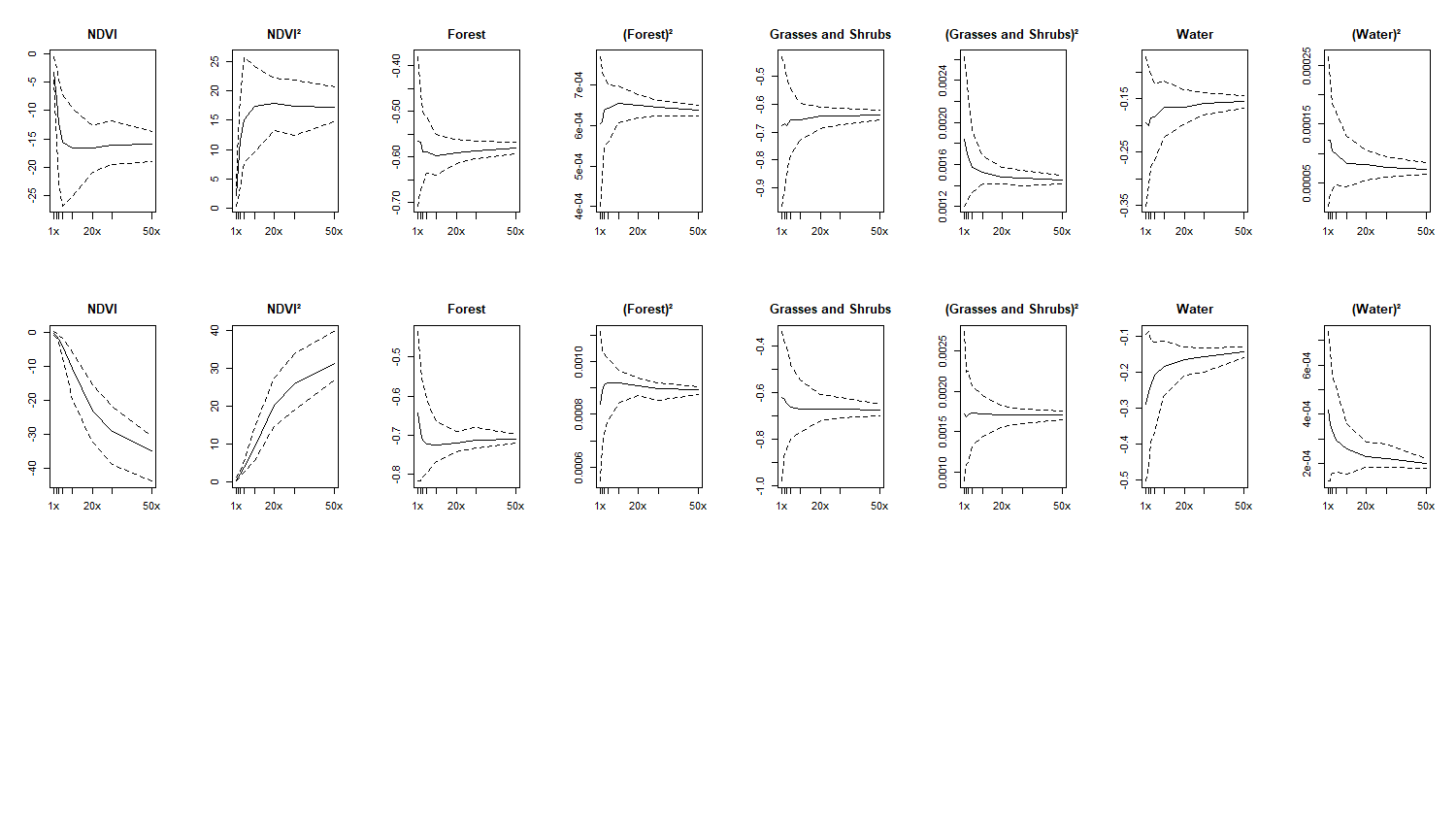


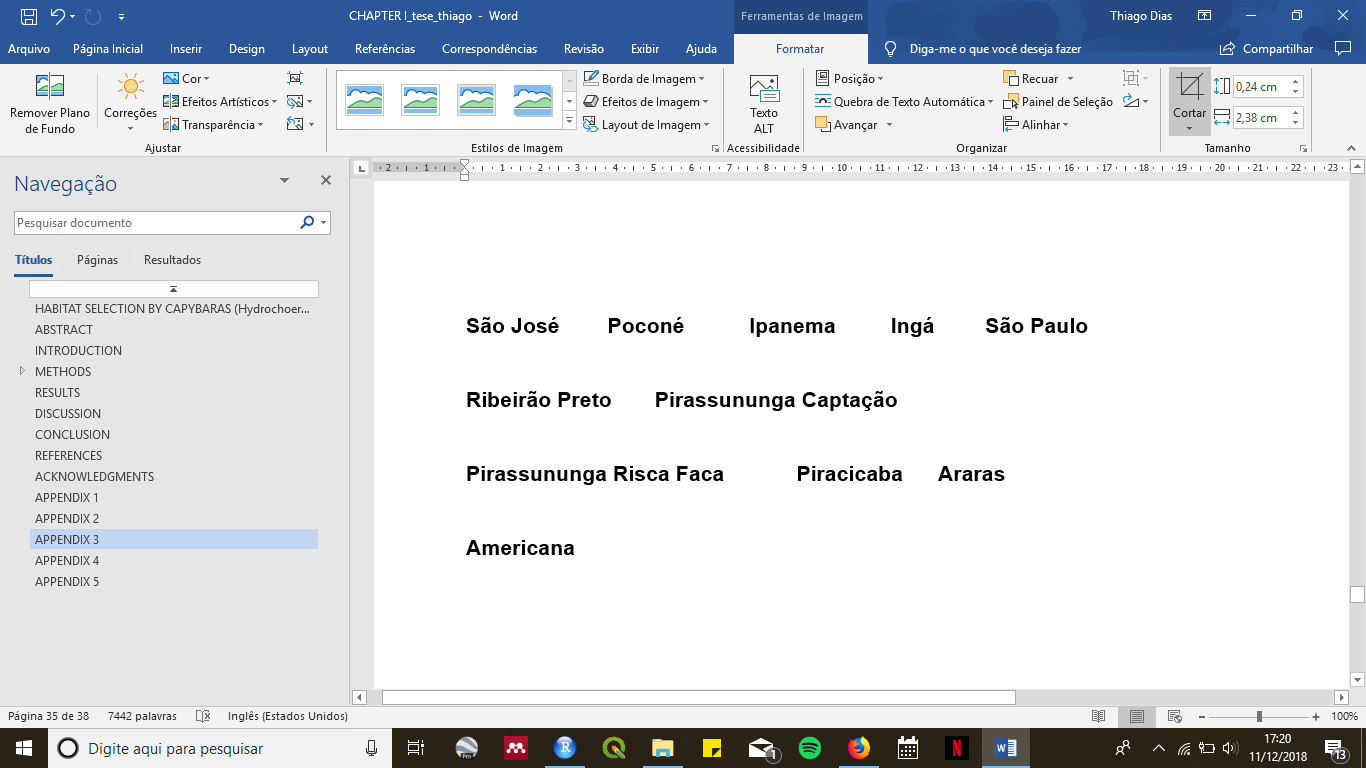

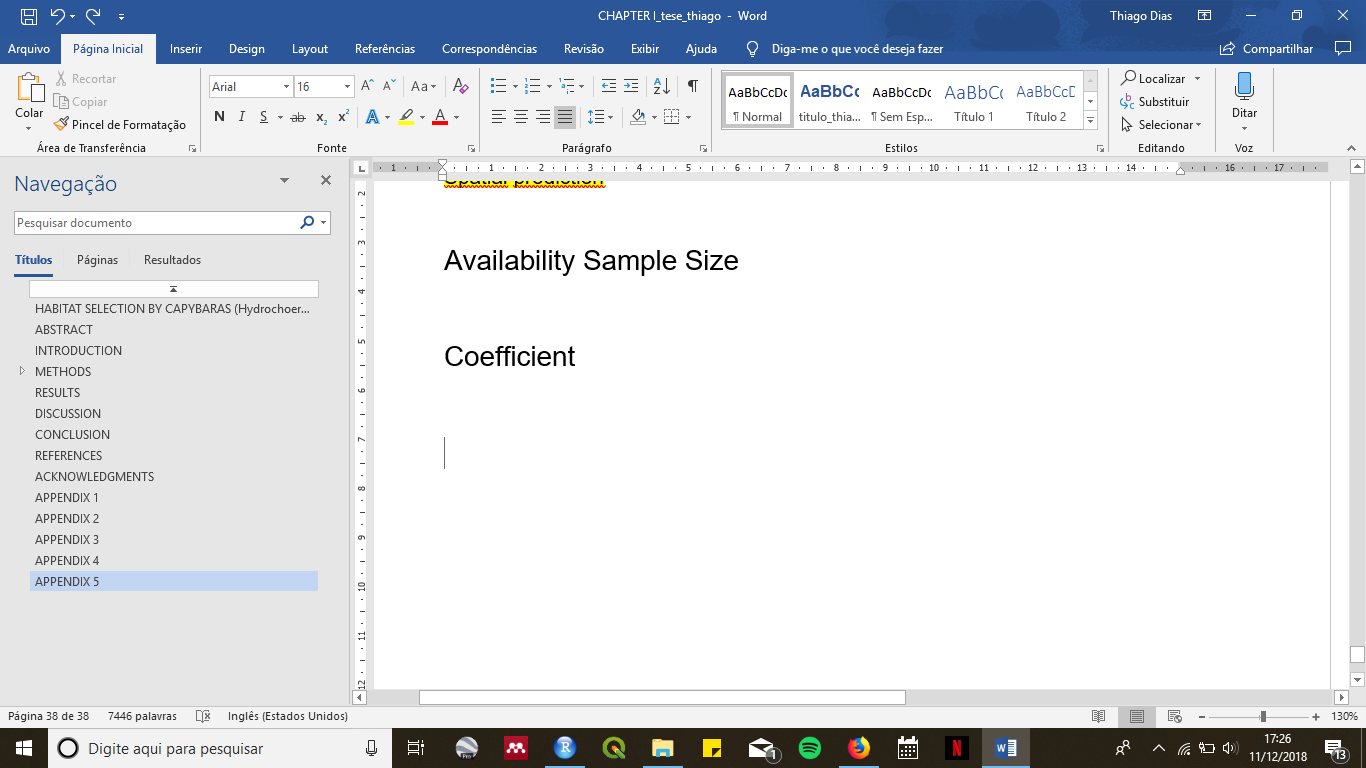

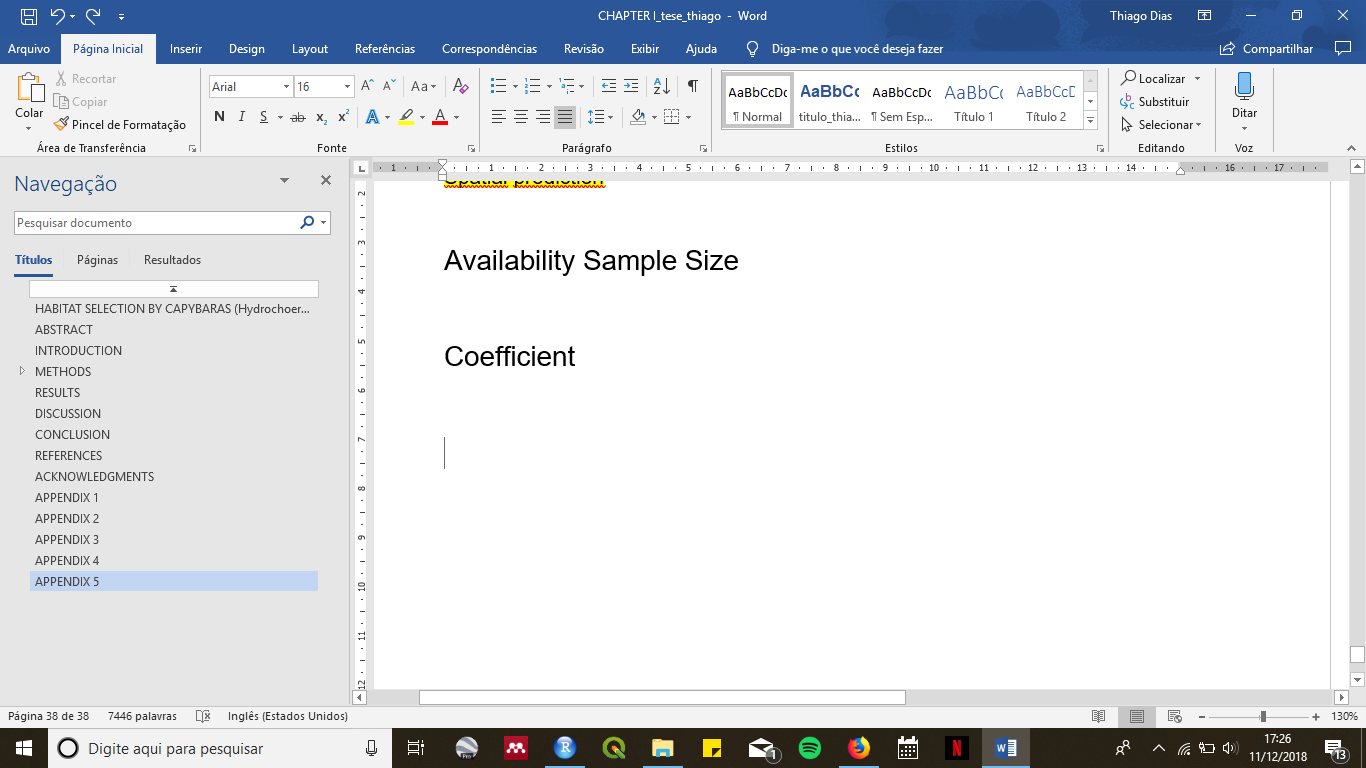

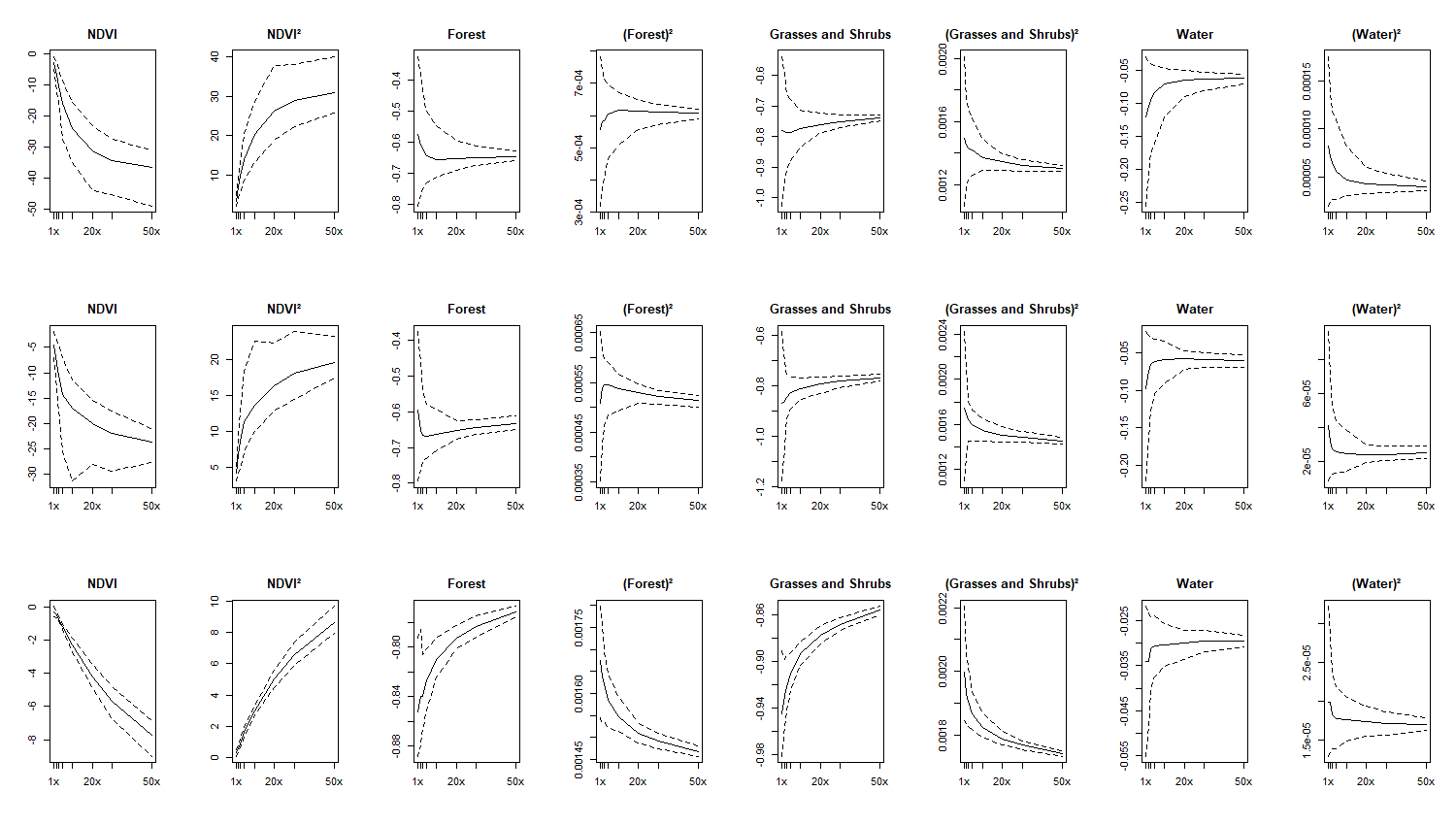

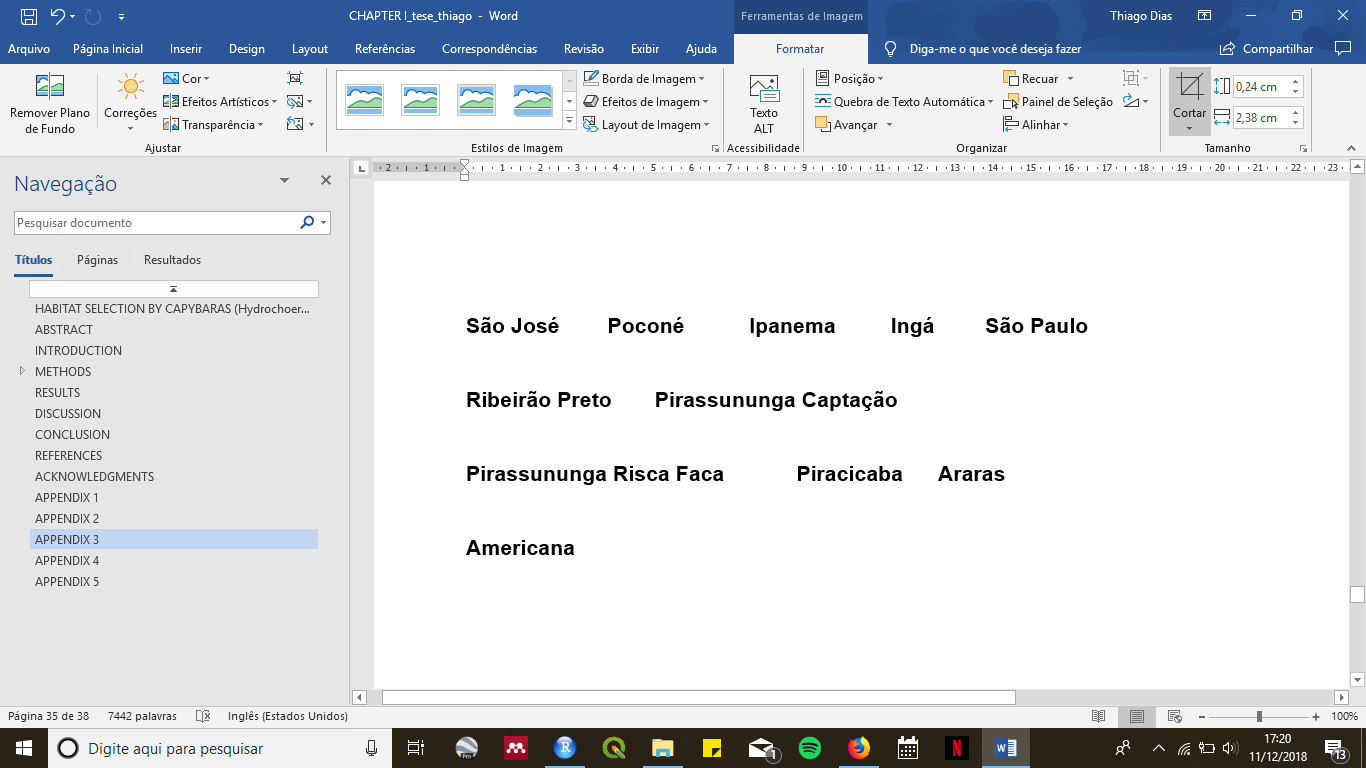

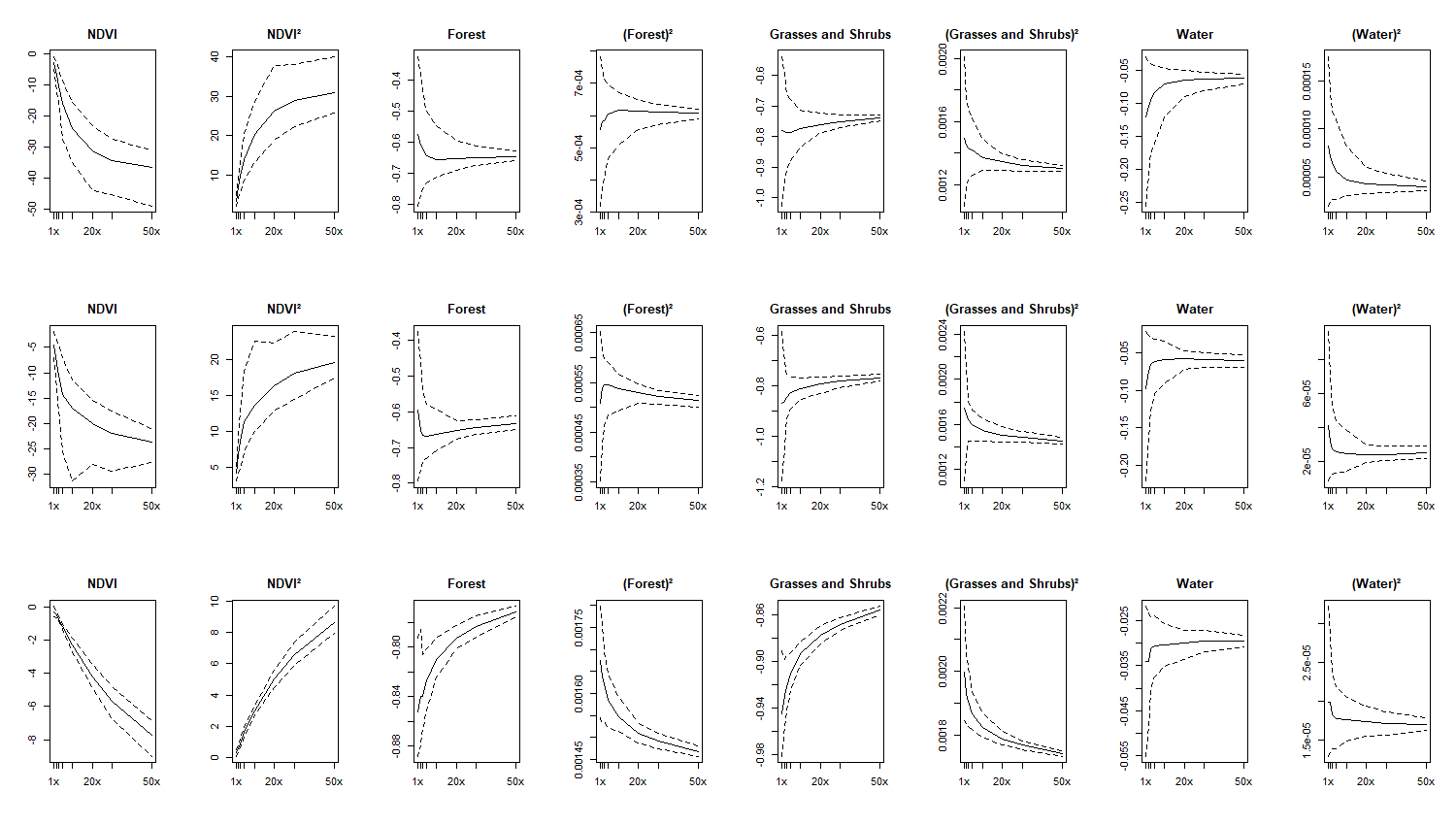


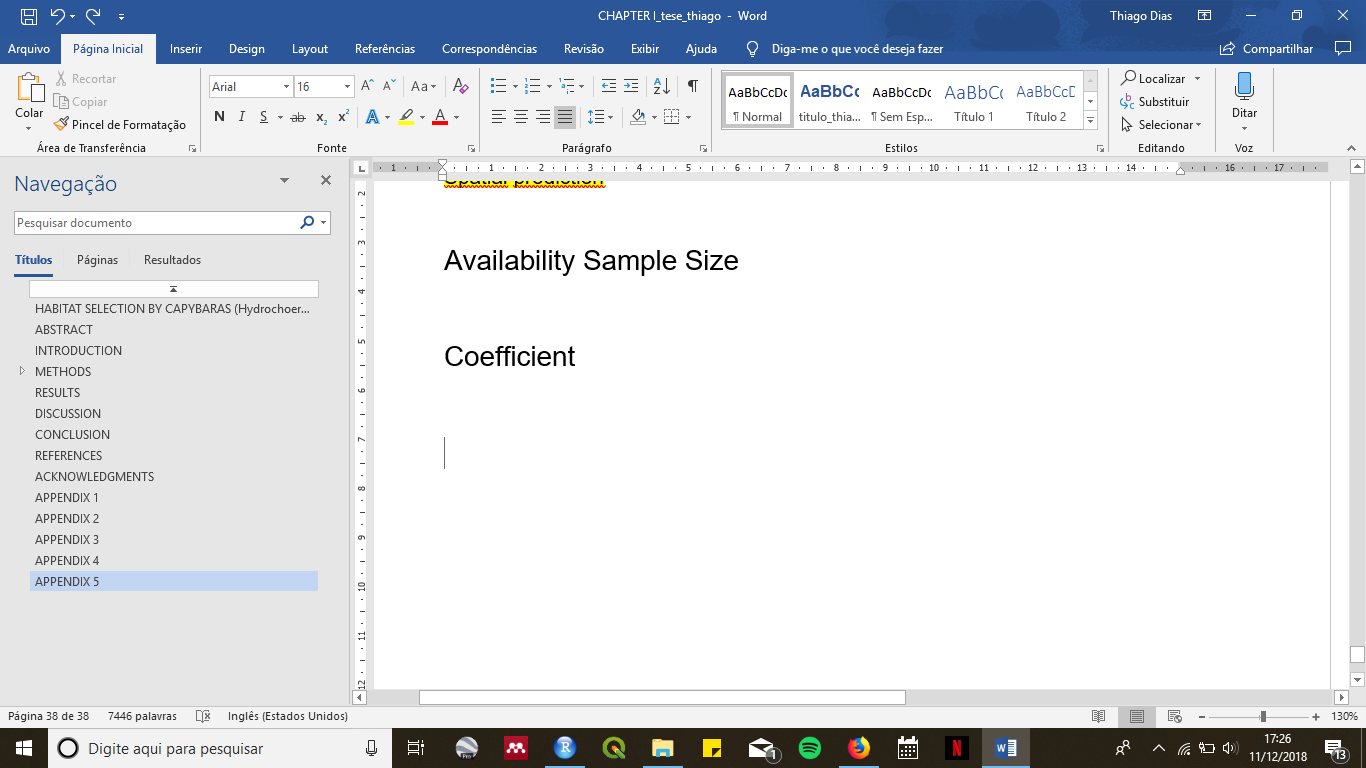

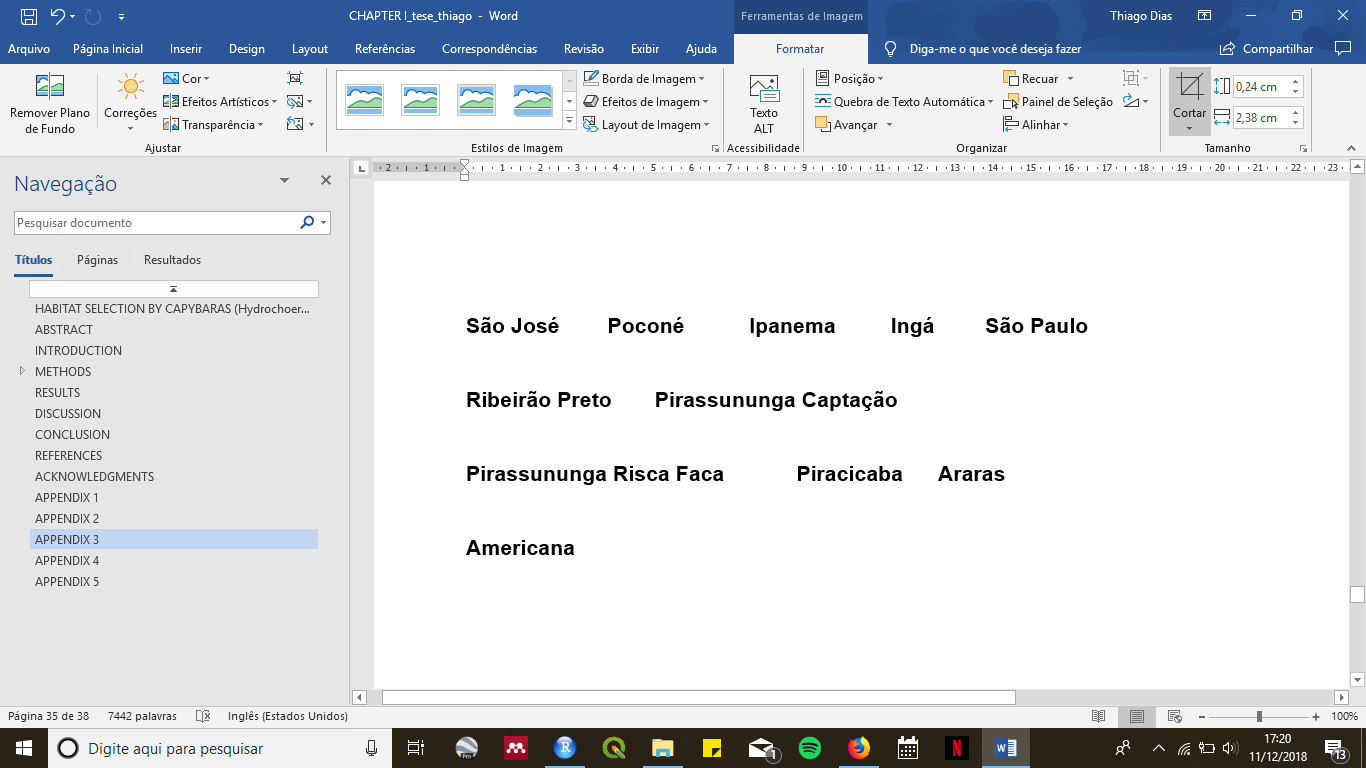

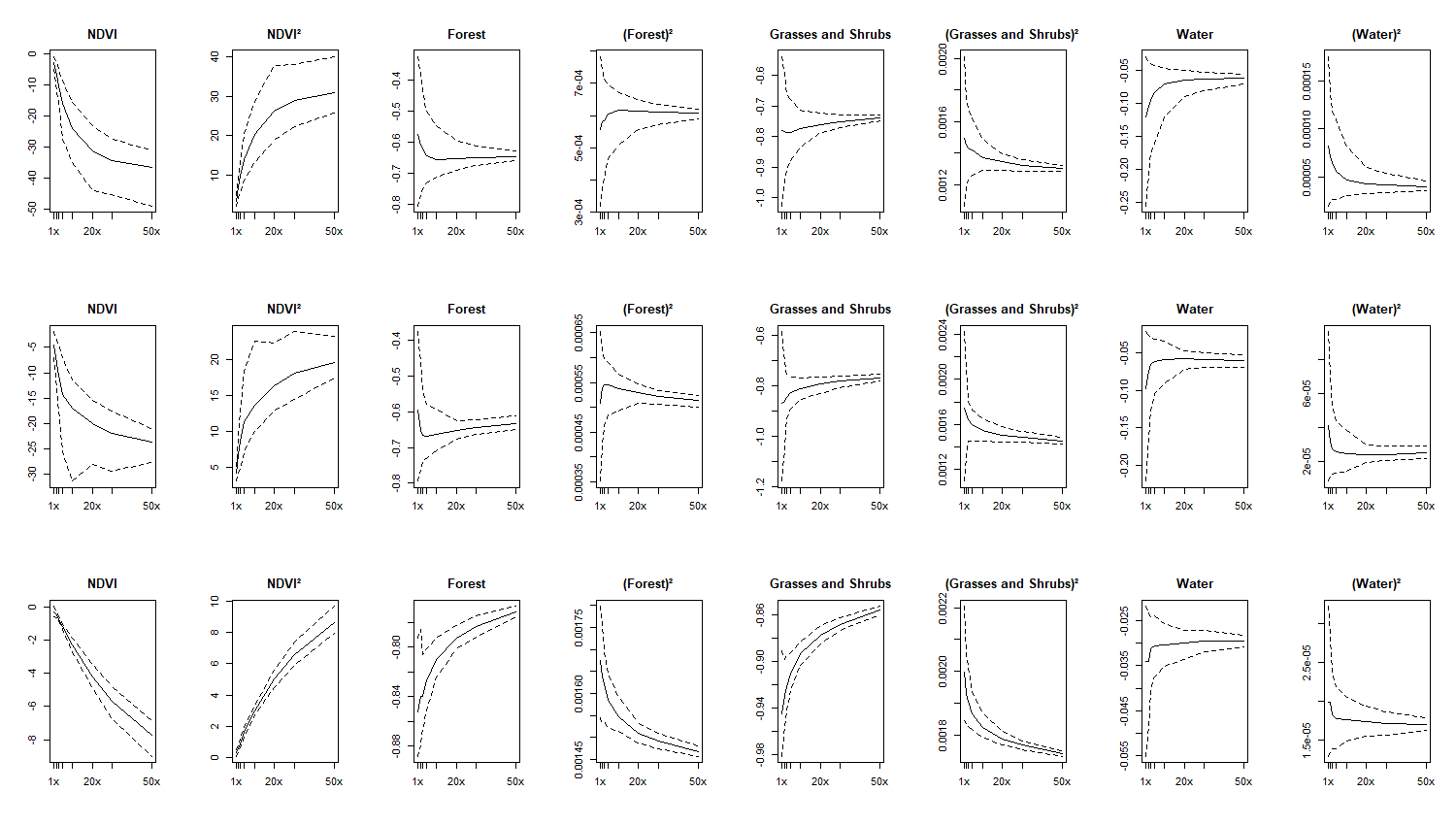


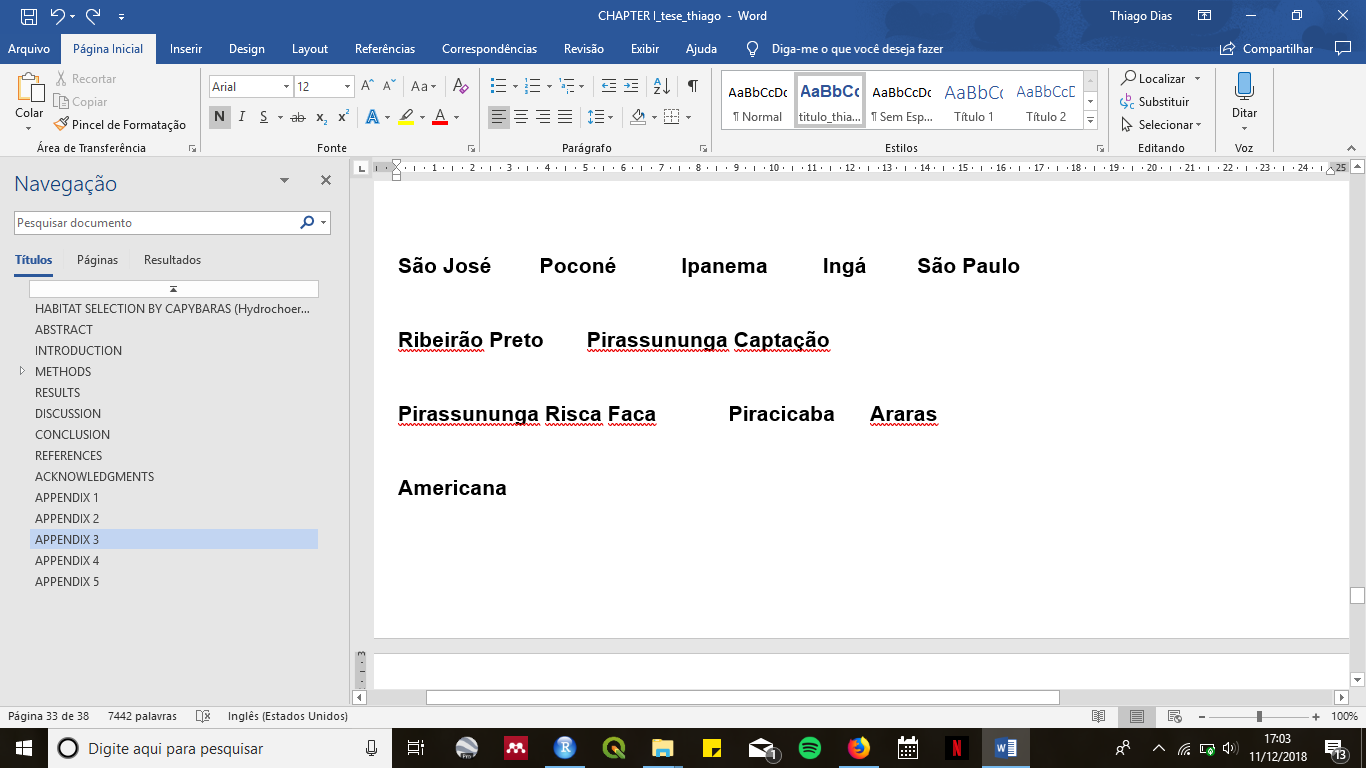

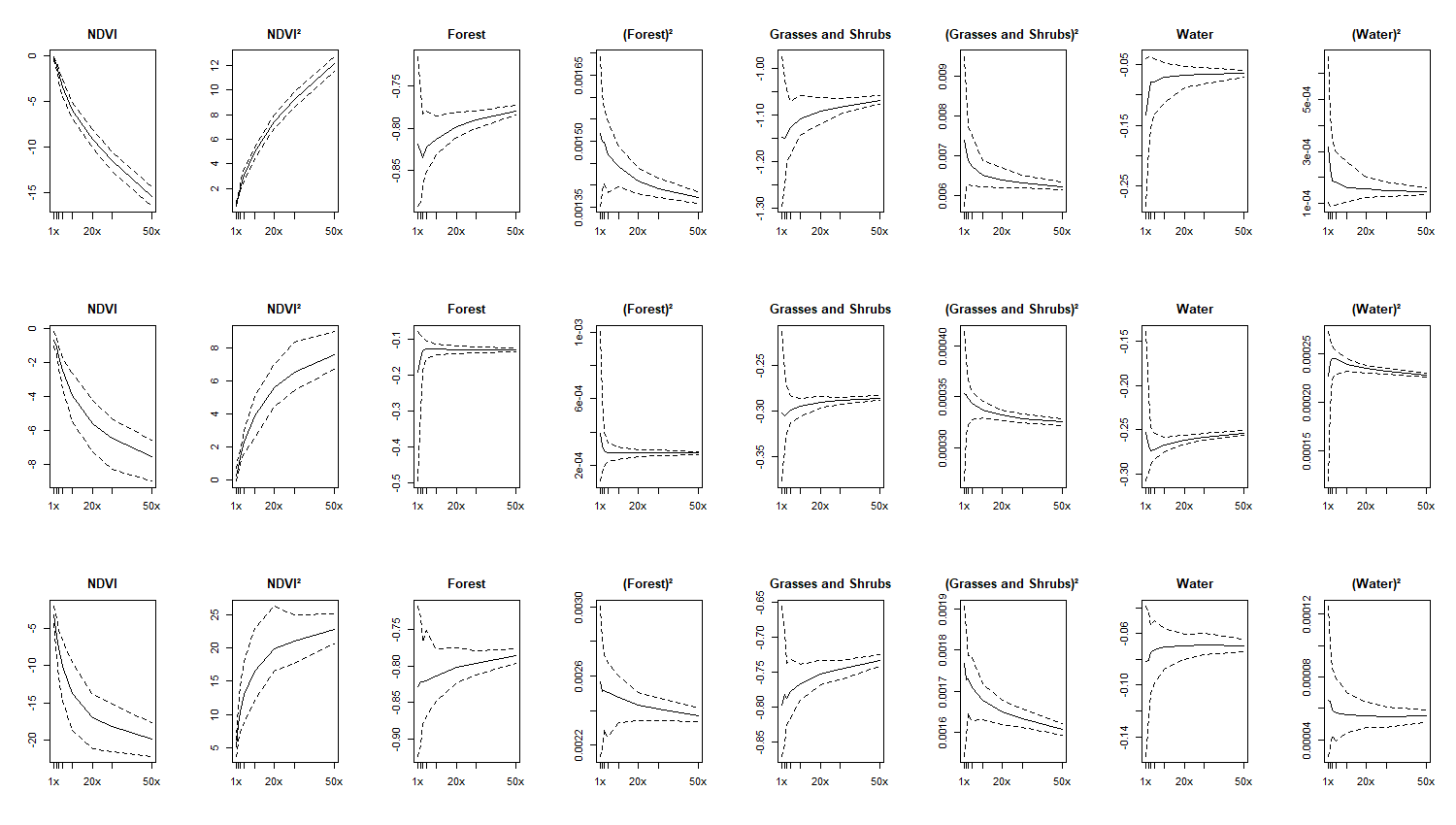


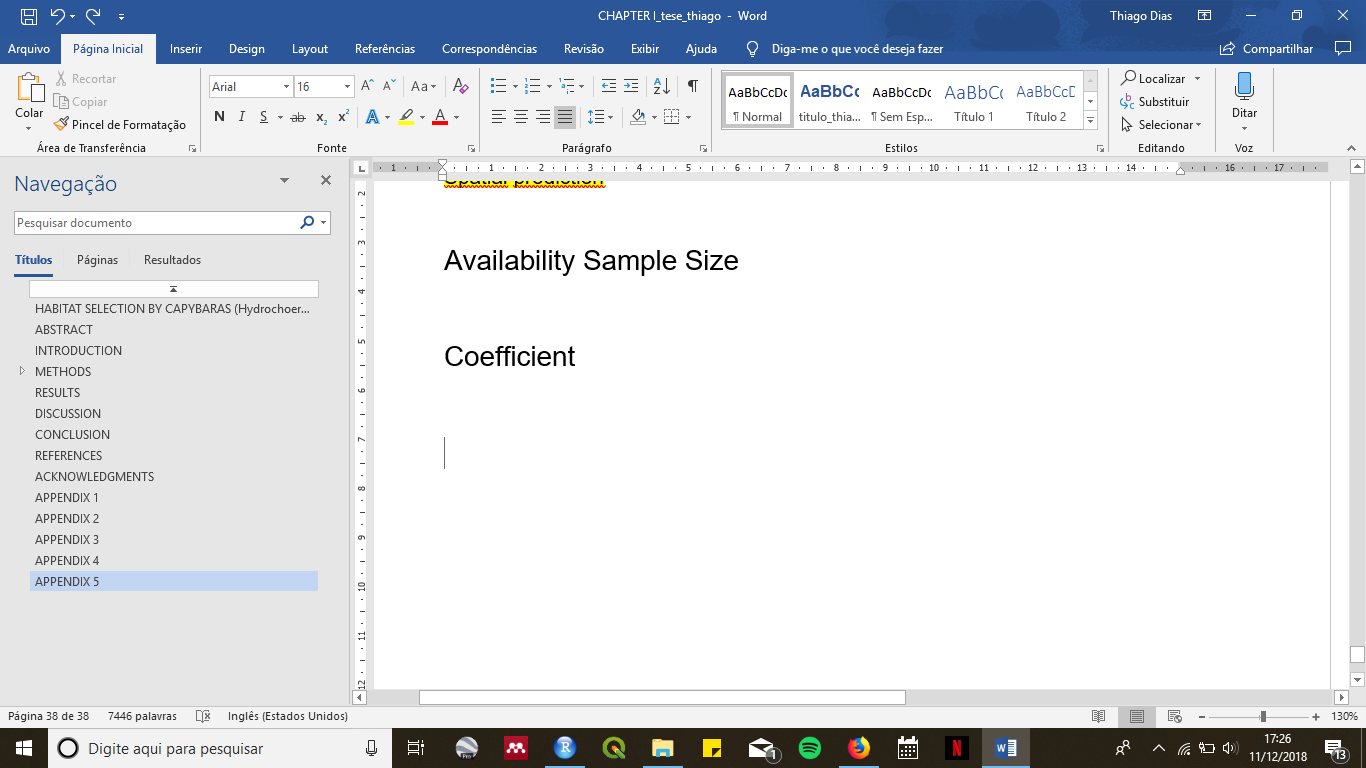

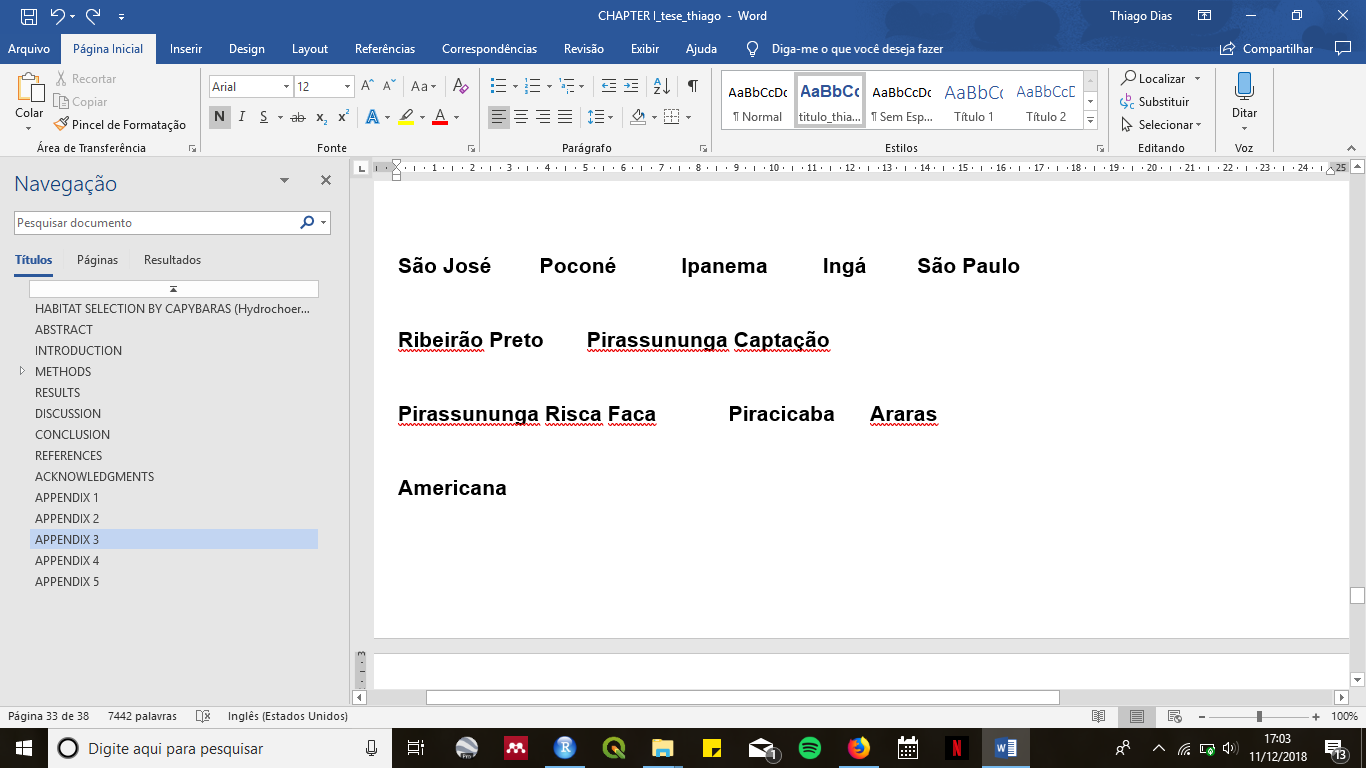

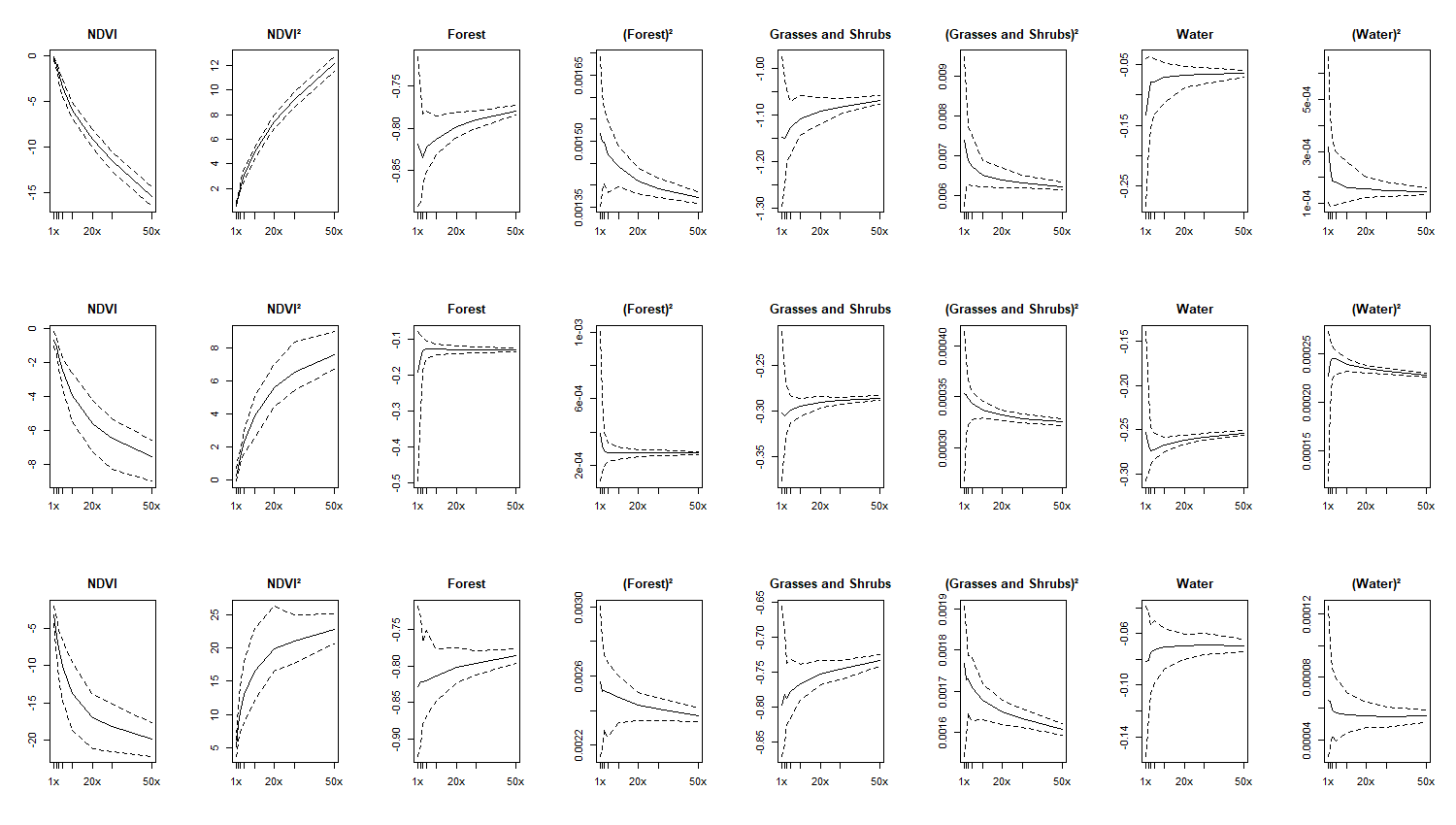


**References**

1. Northrup JM, Hooten MB, Anderson CR, Wittemyer G. Practical guidance on characterizing availability in resource selection functions under a use–availability design. Ecology. 2013; 94: 1456-1463.
