# Supplementary material for: Habitat selection in natural and human-modified landscapes by capybaras (Hydrochoerus hydrochaeris), an important host for Amblyomma sculptum ticks
Source: PLoS One. 2020 Aug 20;15(8):e0229277. doi: 10.1371/journal.pone.0229277 (PMC7444575; doi:10.1371/journal.pone.0229277)
Supplement: S3 Appendix — We used presence-only data to evaluate our top-ranked models’ performance through Spearman rank correlations between area-adjusted frequencies and resource selection functions spatial bins. (DOCX) [file pone.0229277.s003.docx]

# S3 Appendix

Following [1], we randomly selected twenty per cent of presence-only data (GPS-data) to evaluate top-ranked models fit cross-study areas for day- and night periods. Models were evaluated using Spearman rank correlations ($r_{s}$) between area-adjusted frequencies (presence-data frequency) and ten RSF spatial bins [1, 2]. Models with strong positive correlations would be expected to have good spatial predictive performance [1].

Cross-validation results showed good predictive performance for almost all study areas across natural and human-modified landscapes, for both day- and nighttime. Across natural landscapes, Spearman rank correlation coefficients ranged from 0.41 to 1 ($\bar{x} r_{s}=0.85;n=4$) during day periods and from 0.46 to 1 ($\bar{x} r_{s}=0.69;n=4$) during nighttime (Table A). Across human-modified landscapes, values ranged from 0.79 to 0.97 ($\bar{x} r_{s}=0.89;n=7$) for day periods, and from 0.32 to 0.90 ($\bar{x} r_{s}=0.72;n=7$) for night periods (Table A).

Table A. Cross-validation results for habitat selection models across natural and human-modified landscapes for both day- and night periods are displaced by study area. Best models-fit were represented by averaged Spearman rank correlation coefficient (𝑟_𝑠_) close to 1.

| *Study Area* | *Day (*$r_{s}$*)* | *Night (*$r_{s}$*)* |
| --- | --- | --- |
|  | *Natural Landscapes* | |
| São José | 1 | 0.6 |
| Ingá | 1 | 0.4617 |
| Ipanema | 0.4077 | 1 |
| Poconé | 0.9 | 0.7143 |
|  | *Human-modified Landscapes* | |
| Americana | 0.9487 | 0.7714 |
| Araras | 0.9747 | 0.9 |
| Piracicaba | 1 | 0.8536 |
| Pirassununga Risca Faca | 0.6571 | 1 |
| Pirassununga Captação | 0.7857 | 0.4325 |
| Ribeirão Preto | 0.9289 | 0.7667 |
| São Paulo | 0.9266 | 0.3152 |

**References**

1. Boyce MS, Vernier PR, Nielsen SE, Schmiegelow FK. Evaluating resource selection functions. Ecol Model. 2002; 157: 281-300.
2. Stabach JA, Wittemyer G, Boone RB, Reid RS, Worden JS. Variation in habitat selection by white‐bearded wildebeest across different degrees of human disturbance. Ecosphere. 2016; 7: 1-17.
