# Supplementary material for: Habitat selection in natural and human-modified landscapes by capybaras (Hydrochoerus hydrochaeris), an important host for Amblyomma sculptum ticks
Source: PLoS One. 2020 Aug 20;15(8):e0229277. doi: 10.1371/journal.pone.0229277 (PMC7444575; doi:10.1371/journal.pone.0229277)

# S4 Appendix

Figure A. GPS-locations of tracked capybara groups in natural landscapes of the Brazilian Pantanal and human-modified landscapes of São Paulo state, Brazil. Yellow dots represent diurnal locations, whereas blue dots are related to nocturnal GPS-points. The title in each figure section represent the name of study site where capybaras were tracked. Figure was constructed using RGB plots of high-resolution WorldView-2 satellite imagery (DigitalGlobe, Inc.).


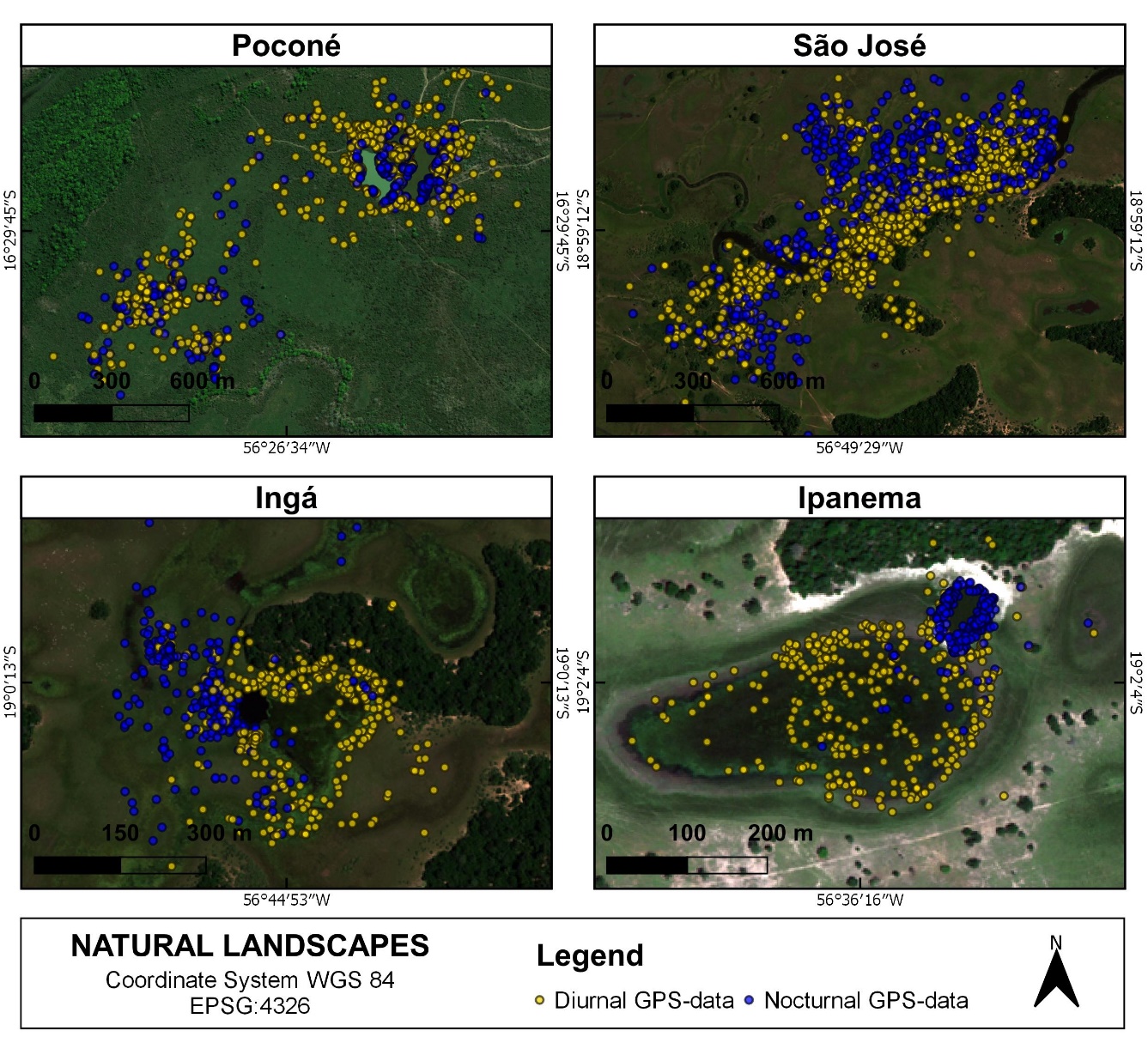


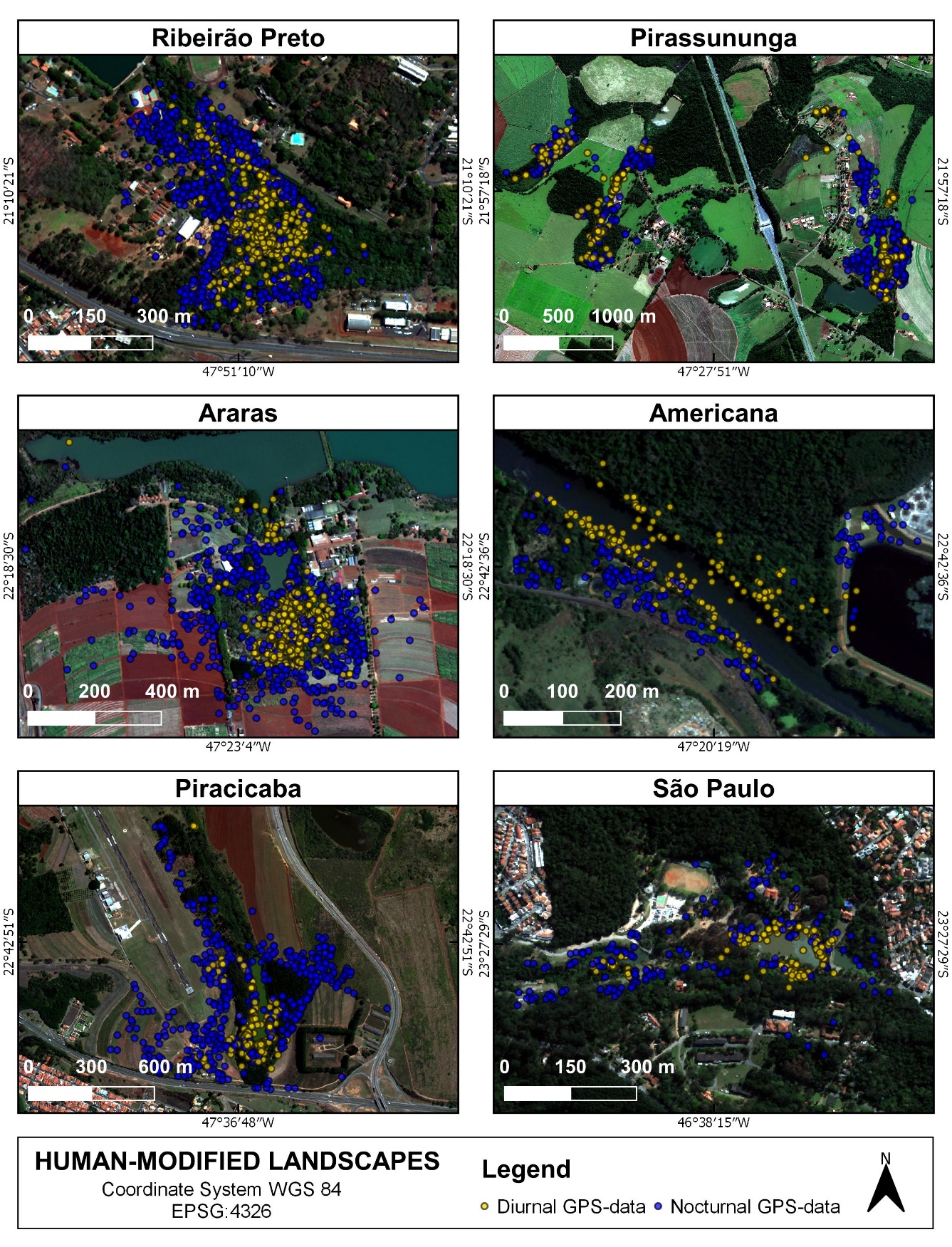

Supplement: S4 Appendix — We plotted diurnal and nocturnal capybara locations at each study area over WorldView-2 satellite imagery. (DOCX) [file pone.0229277.s004.docx]
