# Supplementary material for: Habitat selection in natural and human-modified landscapes by capybaras (Hydrochoerus hydrochaeris), an important host for Amblyomma sculptum ticks
Source: PLoS One. 2020 Aug 20;15(8):e0229277. doi: 10.1371/journal.pone.0229277 (PMC7444575; doi:10.1371/journal.pone.0229277)
Supplement: S1 Table — (DOCX) [file pone.0229277.s005.docx]

# S1 Table

Table S1. Summary table for GPS-tracked capybaras across natural (NLs) and human-modified landscapes (HMLs), including number of GPS locations, duration (in days) and the maximum step length (in meters) by individual across study periods.

|  | *Ind. ID* | *Study Area* | *Start Date* | *End Date* | *Duration (days)* | *GPS-locations* | *MSL* (m)* |
| --- | --- | --- | --- | --- | --- | --- | --- |
| NLs | 1 | São José | 07-27-2015 | 01-30-2018 | 918 | 3939 | 1362 |
|  | 2 | Ingá | 08-22-2017 | 01-30-2018 | 161 | 708 | 592 |
|  | 3 | Ipanema | 08-21-2017 | 01-30-2018 | 162 | 722 | 442 |
|  | 4 | Poconé | 07-21-2016 | 01-30-2018 | 558 | 1719 | 1437 |
| HMLs | 5 | Americana | 06-16-2016 | 09-04-2016 | 80 | 444 | 596 |
|  | 6 | Araras | 09-04-2015 | 11-22-2015 | 445 | 274 | 561 |
|  | 7 | Araras | 12-02-2015 | 05-11-2016 | 161 | 672 | 729 |
|  | 8 | Araras | 06-01-2016 | 07-20-2016 | 49 | 284 | 394 |
|  | 9 | Araras | 09-01-2016 | 12-10-2016 | 100 | 138 | 268 |
|  | 10 | Araras | 10-04-2017 | 01-30-2018 | 118 | 578 | 601 |
|  | 11 | Piracicaba | 10-23-2015 | 02-21-2016 | 121 | 658 | 2703 |
|  | 12 | Piracicaba | 07-13-2016 | 03-03-2017 | 233 | 994 | 1267 |
|  | 13 | Pirass. Ris. Faca | 10-02-2015 | 12-05-2015 | 64 | 281 | 1073 |
|  | 14 | Pirass. Ris. Faca | 06-09-2016 | 04-23-2017 | 318 | 1347 | 689 |
|  | 15 | Pirass. Capta. | 10-02-2015 | 11-04-2015 | 33 | 117 | 805 |
|  | 16 | Pirass. Capta. | 10-17-2016 | 11-21-2016 | 36 | 161 | 743 |
|  | 17 | Pirass. Capta. | 08-10-2017 | 01-30-2018 | 173 | 762 | 1132 |
|  | 18 | Ribeirão Preto | 07-19-2015 | 07-30-2017 | 743 | 3218 | 488 |
|  | 19 | Ribeirão Preto | 07-31-2017 | 01-30-2018 | 183 | 192 | 1162 |
|  | 20 | São Paulo | 10-10-2015 | 01-08-2016 | 90 | 465 | 671 |

*Maximum step length in meters (m)
