# Supplementary material for: Habitat selection in natural and human-modified landscapes by capybaras (Hydrochoerus hydrochaeris), an important host for Amblyomma sculptum ticks
Source: PLoS One. 2020 Aug 20;15(8):e0229277. doi: 10.1371/journal.pone.0229277 (PMC7444575; doi:10.1371/journal.pone.0229277)
Supplement: Attachment — Submitted filename: review_comments.pdf [file pone.0229277.s007.pdf]

Revision of the paper "Human-induced changes in habitat preference by capybaras (Hydrochoerus hydrochaeris) and their potential effect on zoonotic disease transmission"

Dear,

Authors and Editor

Thank you very much for approaching me to review this manuscript. This is an interesting paper in which the authors quantified the habitat use of capybaras by means of Resource Selection Function of GPS-tracked capybaras. They aimed to compare a degraded landscape vs a more conserved forested ecosystem and found out that capybaras tend to be more restricted to the forest in degraded landscapes than those in natural areas, most likely to avoid contact with humans. They use these results to develop hypotheses regarding the repercussion of this behavior on the spreading of a common disease in Brazil.

Minor revisions

L47: I would be more careful when making the statement that capybara is a "conflict species", please reword. You can say of course that the species can cause problems in some areas (which ones), but such an overarching adjective is not appropriate for any species. Besides, the argument after the sentence makes me think that you are talking about a different kind of issue, the Human Wildlife Conflict (HWC) is usually well define, please revise this and reword accordingly.

L49: all cases around the world? The capybara is only Neotropical, even other regions issues are caused by capybara? What is "has been implicated", can you be more specific?

L54: higher?
L57: can you provide the direction of the effect of the most important variables? (is water = distance to water?)

*the abstract is focus on the resource selection and we don´t really understand the link with the disease transmission which is included as a main topic in the title, can you comment on that, would be great.

L65: just in case, not all species are forced to adapt to HDL, actually many others also show preferences and look for human infrastructures, but not the topic of the paper…
L67: spatial ecology is too broad, and actually makes us think about the discipline, can you be more specific, e.g. distribution, occupancy, habitat use?

L81. Lethal to who? Cattle, sheep, horses? All domestics?
L90. It just sounds weird to put the wildebeest to compare capybaras, they are completely different species/groups, I would change the ref
L91. The first prediction does not really sound like a prediction, please be more specific of what you expect (e.g. how variation?), it seems that it is contained in b so maybe there is no A? (please reword "must show" for something more formal)
L105. 11 groups mean you tracked all members of each group?, please rephrase accordingly, would be good to have an indication of the number of individual per group, that also tends to

affect animal movements. I don´t think this part fit well into “study area” and rather confuses the reader. It sounds more like an analysis or any other section. Please move accordingly and focus just on the area itself, the variables can go in other section.

L110: please clarify (in general) how this index works

L118: so you did tag several individual within each group? Then you get a mean dispersal distance for the group, and you use that area to calculate the HFI? When do you sum up the values. This part is a bit confusing, please reword. Why summing is better than averaging? Please justify so the reader understands better (for what is stated in L20 it seems to be a mean actually)

Please double check the English and grammar in a few places.

Lastly, I know the main purpose of the paper was not to study the transmission of the disease itself, but I found it difficult to link the actual study (RSF with capybaras) to the spread of the BSF. Although the arguments are strong and well supported by references, it would have been great if you had quantified something from the animals that were captured. If you have more information, that would make the suggestions stronger (for example, an estimation of the actual number of infested animals in your groups), it would contribute to make the hypothesis more attractive.

Best wishes, I look forward to seeing this paper published.
