# Supplementary material for: Habitat selection in natural and human-modified landscapes by capybaras (Hydrochoerus hydrochaeris), an important host for Amblyomma sculptum ticks
Source: PLoS One. 2020 Aug 20;15(8):e0229277. doi: 10.1371/journal.pone.0229277 (PMC7444575; doi:10.1371/journal.pone.0229277)
Supplement: Attachment — Submitted filename: Response to Reviewers.docx [file pone.0229277.s008.docx]

26 April 2020

Dr Daniel Becker

Academic Editor
PLOS ONE

Ref: PONE-D-20-02991

Title: Human-induced changes in habitat preference by capybaras (Hydrochoerus hydrochaeris) and their potential effect on zoonotic disease transmission

Journal: PLOS ONE

Dear Dr Becker,

We are submitting the revised version of our manuscript “Habitat selection in natural and human-modified landscapes by capybaras (*Hydrochoerus hydrochaeris*), an important host for *Amblyomma* *sculptum* ticks” (new title), which was modified based on reviewers’ comments and suggestions.

Thank you.

Best regards,

Thiago C. Dias

Corresponding author on behalf of co-authors

**AUTHORS’ RESPONSES**

**Academic Editor**

Thank you for the comments and suggestions, they were all very important to improve the quality of our work. The manuscript was modified and we changed the sentences where the link between capybara habitat selection and BSF was not clear. We also replaced the title of the manuscript to “Habitat selection in natural and human-modified landscapes by capybaras (*Hydrochoerus hydrochaeris*), an important host for *Amblyomma* *sculptum* ticks”. The problem with the figure copyright (Fig 1) was also resolved, we removed the layer needing copyright.

**RESPONSES TO REVIEWERS #1, #2 and #3**

(line numbers below refer to the “clean text version”; not the marked text version)

**Reviewer #1:**

Lines 66-68, sentence beginning “Across these landscapes”: This sentence could use some elaboration as it touches on several large concepts (human disturbance, wildlife responses, zoonotic disease transmission). Putting in some concrete examples may help here.

Response: We accepted this suggestion and added new information to the paragraph.

Please provide more detail on the second paragraph of the introduction. For example, it isn’t mentioned that capybaras are found in South America, and it isn’t clear if their populations are increasing across all of their distribution or just certain places.

Response: We added information about capybara distribution and habitat use as suggested. We also changed the beginning of third paragraph [revised manuscript] to specify that capybara populations are growing only over human-modified landscapes.

Lines 85-87: Please expand on this sentence—how might human-driven variation in capybara habitat selection affect BSF dynamics? Could you provide more details from References 16-19 for readers who aren’t as familiar with the previous work that has been done?

Response: We removed the sentence from here and also provided more details from references 16-19 in the fourth paragraph [revised manuscript].

Table 1: Was there a reason that there wasn’t a fifth model with just NDVI and NDVI^2?

Response: We haven’t created models inclusive only of NDVI because we only had one satellite scene per study area, so we think NDVI by itself would not be a good model. On the other hand, NDVI was important to improve our land cover classification and as a part of the other models.

Tables 2 and 4: Given that the full model was by far the best for natural and human-modified habitats, in both day and night, I think these tables could be moved to the supplementary material. It would also be preferable to rank the models by delta AIC.

Response: Thank you for your comment but we think that it is important to keep the table in the main manuscript since the results in there were important to subsequent analysis. We ranked the models by delta AIC as suggested.

Table 3: This might be easier to interpret as a figure (e.g. forest plot) rather than a table.

Response: We tried to plot it as a figure and the visualization wasn’t good so we decided to keep it as a table. Individual plots can be found in Figures 2 and 3.

Lines 353-354: Are there direct observations of capybaras moving to avoid people, traffic, etc?

Response: Here we added a personal communication and a reference of capybaras using forests to avoid human contact.

Lines 372-374: I found that this conclusion was not supported by the data. Given that there was no sampling of ticks and R. rickettsia from the capybaras, I don’t think the authors can conclude definitively that human-driven changes in capybara habitat selection have influenced BSF spread. Providing more details from previous work such as Reference 19 could be helpful here. The authors could also qualify their language to clarify that they did not directly study the role of capybaras in BSF spread.

Response: Thank you for your suggestion, we removed this sentence and added more information about ref 19, a study performed by colleagues in the same study areas and with the same capybara populations that we worked.

The second to last paragraph of the Discussion felt like it did not add new information, and might belong more in the Introduction.

Response: We removed the paragraph.

*All minor comments were also accepted and the text was modified as suggested by the reviewer.*

**Reviewer #2:**

I think that the wording “and their potential effect on zoonotic disease transmission” implies that you measured something pertaining to pathogen transmission. Something like “Habitat selection in human-modified landscapes by capybaras, an important host for Amblyomma sculptum ticks” would be more accurate to the contents of the analysis

Response: Thank you for this comment, we changed the title to “Habitat selection in natural and human-modified landscapes by capybaras (*Hydrochoerus hydrochaeris*), an important host for *Amblyomma* *sculptum* ticks”, as you have suggested.

L85-87: I would suggest adding more support for how capybara habitat selection in human modified areas would impact BSF epidemiology. What shifts in habitat selection would increase human risks

Response: As the link wasn’t clear, we removed the paragraph and added more information about the relationship between capybara and *A. sculptum* ticks.

Figure 1: Does the gray shading in rightmost panels indicate topography? I think it would be more useful to show the human footprint layer or land cover data instead.

Response: Thank you for your suggestion, we have now a clean figure focused on show the location of our study areas. Topography layer was removed

L180: what was your definition of diurnal and nocturnal locations based on the time on sunrise and sunset? Did a location have to be within a certain number of hours after sunrise and before sunset to be considered diurnal?

Response: We defined day as the period between sunrise and sunset, and night as the period between sunset and sunrise. We changed the text to make it more clear.

It would be helpful to see a map figure with capybara locations on it.

Response: We agree, but unfortunately, we have too many study areas located far away from each other, so it was impracticable to plot capybara locations in a single plot. If we did plot locations, we would need several single plots to do that. If you think that is necessary, we can add this in supporting information.

Table 3: please report the random effect variance

Response: We now reported the random effects in Table 3.

L353: could the increased selection for forested areas be also simply a function of lower forest availability in human modified landscapes? Highlighting that capybaras selected more strongly for forested areas during the day vs the night when capybaras in natural landscapes did not show that difference with time period would strengthen your argument.
Response: We don’t think that this is what is happening. Pantanal is not a woodland dominated landscape, being characterized by a mosaic of upland vegetation and seasonally flooded areas as we specified in “Methods - Study Area”. In addition, forest availability is not equal in our human-modified landscapes, and we do have relatively forested areas across them. We accepted your suggestion about highlighting capybara forest selection (day vs night) in human-modified landscapes, the text was modified, thank you.

L355: this is not clear. What do you mean by the relationship being more pronounced in open areas? Was the location of the animal within the study area taken into account?

Response: Here we were talking about human disturbance being more pronounced in open areas of our study sites during daytime. Since there is no or low human activities inside forests or in the water, we decided to specify where activities are pronounced. We changed the text in order to make it clearer.

L359 – 364: this information about A. sculptum habitat preferences is important for your rationale and should be in the introduction.

Response: Thank you, we added this information in the introduction.

I think there should be some caveats in the discussion section acknowledging the small sample size for capybaras in the natural landscapes. It is difficult to generalize based on 4 individuals.

Response: We added a sentence in the last paragraph of our discussion about this subject. Even if there are only 4 individuals tracked in natural landscapes, they all show relatively large datasets and track periods, which increases the reliability of our data.

L416: Did you compare the use of forested areas between these landscapes? I thought the interpretation would be that capybaras selected for areas closer to forests in human modified landscapes than natural landscapes.

Response: Thanks for your comment, we changed the text as suggested.

*All minor comments were also accepted and the text was modified as suggested by the reviewer.*

**Reviewer #3:**

L47: I would be more careful when making the statement that capybara is a “conflict species”, please reword. You can say of course that the species can cause problems in some areas (which ones), but such an overarching adjective is not appropriate for any species. Besides, the argument after the sentence makes me think that you are talking about a different kind of issue, the Human Wildlife Conflict (HWC) is usually well define, please revise this and reword accordingly.

Response: Thank you for your suggestions. We removed the term “conflict species” and added examples of HWC that capybaras are involved as well.

L49: all cases around the world? The capybara is only Neotropical, even other regions issues are caused by capybara? What is “has been implicated”, can you be more specific?

Response: Here we were talking about capybaras being linked to the most lethal tick-borne disease in the world, occuring in South America. We changed the text to make more clear.

L57: can you provide the direction of the effect of the most important variables? (is water = distance to water?)

Response: We provided the direction of the effects in Table 3. We also changed “water” to “distance to water” as suggested.

*the abstract is focus on the resource selection and we don´t really understand the link with the disease transmission which is included as a main topic in the title, can you comment on that, would be great.

Response: We changed the title to “Habitat selection in natural and human-modified landscapes by capybaras (*Hydrochoerus hydrochaeris*), an important host for *Amblyomma* *sculptum* ticks”, as suggested by another reviewer. We also modified the discussion to highlight the link between shared preferences in habitat selection by capybaras and the tick *A. sculptum*, a vector for the bacterium that causes the BSF. We tried to be more careful when making statements on the link between capybaras and BSF transmission as well.

L65: just in case, not all species are forced to adapt to HDL, actually many others also show preferences and look for human infrastructures, but not the topic of the paper…
 Response: Thank you for your comment!

L67: spatial ecology is too broad, and actually makes us think about the discipline, can you be more specific, e.g. distribution, occupancy, habitat use?

Response: We added more specific references here.

L81. Lethal to who? Cattle, sheep, horses? All domestics?

Response: Lethal to humans, we added this information to the text.

L90. It just sounds weird to put the wildebeest to compare capybaras, they are completely different species/groups, I would change the ref

Response: We removed this reference and rephased the sentence.

L91. The first prediction does not really sound like a prediction, please be more specific of what you expect (e.g. how variation?), it seems that it is contained in b so maybe there is no A? (please reword “must show” for something more formal)

Response: Thank you for the suggestion, we reformulated and merged the two predictions.

L105. 11 groups mean you tracked all members of each group?, please rephrase accordingly, would be good to have an indication of the number of individual per group, that also tends to affect animal movements. I don´t think this part fits well into “study area” and rather confuses the reader. It sounds more like an analysis or any other section. Please move accordingly and focus just on the area itself, the variables can go in other section.

Response: Capybaras were tracked with a collar per group; therefore, more than one animal was tracked in certain groups but there was no simultaneously tracking inside the same group. We reformulated the sentence to make it clearer and also changed it to the section “Capybara capture and collaring”.

L110: please clarify (in general) how this index works

Response: We added information about HFI as suggested.

L118: so you did tag several individual within each group? Then you get a mean dispersal distance for the group, and you use that area to calculate the HFI? When do you sum up the values. This part is a bit confusing, please reword. Why summing is better than averaging? Please justify so the reader understands better (for what is stated in L20 it seems to be a mean actually)

Response: We calculated the mean GPS coordinate by individual and used capybara mean dispersal distance found by colleagues [ref 27] as a radius to generate individual buffers. Buffers belonging to the same study areas were merged and we calculated the mean HFI within them. The text was reformulated in order to make it clearer.

Lastly, I know the main purpose of the paper was not to study the transmission of the disease itself, but I found it difficult to link the actual study (RSF with capybaras) to the spread of the BSF. Although the arguments are strong and well supported by references, it would have been great if you had quantified something from the animals that were captured. If you have more information, that would make the suggestions stronger (for example, an estimation of the actual number of infested animals in your groups), it would contribute to make the hypothesis more attractive.

Response: Thank you for this comment. The discussion was reformulated and we added information about a recent study carried out with the same animals studied here and in the same study areas [ref 29]. We also provided new information about environmental tick density and tick abundance in capybaras across natural and human-modified landscapes that we studied. The link between capybaras and ticks was also highlighted in discussion, and we removed sentences linking capybaras to BSF spread.
