# Supplementary material for: Habitat selection in natural and human-modified landscapes by capybaras (Hydrochoerus hydrochaeris), an important host for Amblyomma sculptum ticks
Source: PLoS One. 2020 Aug 20;15(8):e0229277. doi: 10.1371/journal.pone.0229277 (PMC7444575; doi:10.1371/journal.pone.0229277)
Supplement: Attachment — Submitted filename: Response to Reviewers.docx [file pone.0229277.s009.docx]

27 July 2020

Dr Daniel Becker

Academic Editor
PLOS ONE

Ref: PONE-D-20-02991

Title: Habitat selection in natural and human-modified landscapes by capybaras (*Hydrochoerus hydrochaeris*), an important host for *Amblyomma* *sculptum* ticks.

Journal: PLOS ONE

Dear Dr Becker,

We are submitting the revised version of our manuscript “Habitat selection in natural and human-modified landscapes by capybaras (*Hydrochoerus hydrochaeris*), an important host for *Amblyomma* *sculptum* ticks”, which was modified based on reviewer #3 comments and suggestions.

Thank you.

Best regards,

Thiago C. Dias

Corresponding author on behalf of co-authors

**AUTHORS’ RESPONSES**

**Academic Editor**

Thank you for your patience and support during this whole process. We had accepted all minor comments and suggestions from reviewer #3 and modified the text according to them.

**RESPONSES TO REVIEWER #3**

(line numbers below refer to the “clean text version”; not the marked text version)

**Reviewer #3:**

I am confused about figure 1. In the first manuscript you provided a much better figure. The current is not in a good resolution, but most importantly it is not informative. Please use the first figure for publication. Over this map, I would highly recommend putting a landuse/cover layer on the orange area (HDL), as it would give us a clear picture of the patchiness and landuses across that area. The green section must be just pantanal, but would be good to see the other elements included under this category (e.g. riparian forest). Further you are providing too much details about how you constructed the map (e.g. using R etc), that should stay in the methods section. Not needed for the caption. Would it be possible to include the locations of the animals at each location? (would be a very nice supplementary data).

Response: As suggested, we added a land cover layer to natural and human-modified areas and removed unnecessary information about figure 1 construction. We also believe that this will provide a better general overview on the differences between our study sites. We added a new section to supplementary material containing GPS-locations of each tracked group of capybaras. Thank you for your suggestions.

In the study area section there is still a lot of information that is more concerned to the analysis itself. For example, the paragraph describing how they assess the level of human disturbance must be moved to the adequate position in analysis. In study area just provide the geographical context and landscape characteristics so we understand what is human dominated vs natural etc. (paragraph of L138 is also data anaysis, when you move it, please state at the beginning why you did what you are describing e.g. “to xxx we calculated the mean….as currently stants it is not very clear, e.g. so the index also need the GPS coordinates then? Seems like but if the reader is told at the beginning of the paragraph then it would make more sense. Move parag L145 to the end.

Response: We moved the paragraph to “Habitat data” section. We also started the paragraph in L138 stating at the beginning why we performed the analysis. Paragraph L145 was also moved to the end of “Study area” section.

For the discussion it might be worth mentioning the results of Pardo et al. 2020 (Land Management strategies can increase oil palm use by mammals in Colombia; https://www.nature.com/articles/s41598-019-44288-y). They found that “habitat use was 3.6 times greater in riparian forest than in oil palm, and decreased with increasing distance to nearest forest patch, with detections never occurring further than 357 m from remnant forest”. This supports your findings of higher preferences for forest interior in HDL and areas close to forests. This could help to support your personal observation in L399 or L425 or elsewhere.

Response: Thank you for this paper! We added this information to our discussion.

*All minor comments were also accepted and the text was modified as suggested by the reviewer.*
